# A Cluster Randomised Trial of a School‐Based Universal Intervention Program for Middle School Students' Sleep and Related Outcomes

**DOI:** 10.1111/jsr.70123

**Published:** 2025-06-18

**Authors:** Yanchen Zhang, Kayla T. Johnson, Kyla Wahlstrom, Rachel Widome, Corinne Hamlin, Andrew J. Barnes

**Affiliations:** ^1^ Department of Psychological & Quantitative Foundations University of Iowa Iowa City Iowa USA; ^2^ Division of Epidemiology and Community Health University of Minnesota Minneapolis Minnesota USA; ^3^ Organizational Leadership, Policy, and Development University of Minnesota Minneapolis Minnesota USA; ^4^ Clinical and Translational Science Institute, University of Minnesota Minneapolis Minnesota USA; ^5^ Department of Pediatrics University of Minnesota Medical School Minneapolis Minnesota USA

**Keywords:** adolescent, middle school, sleep, theory of planned behaviour, universal prevention

## Abstract

Most middle school students have entered or started entering adolescence, which involves rapid and significant neurodevelopmental changes associated with their sleep. International literature indicates that myriad school‐ and health‐related outcomes are influenced by adolescents' sleep. As a multidisciplinary team, the authors designed a theory‐informed universal intervention program (Sleep to Enhance Educational Performance in Schools; *SLEEPS*) that leveraged the social‐cognitive influences of peers, caregivers and teachers on adolescents' sleep hygiene. This cluster randomised trial was designed to explore the effectiveness of *SLEEPS* on middle school students' sleep and related outcomes. Eight middle schools in the US (*n*_teacher_ = 8, *n*_student_ = 104) were randomly assigned to the treatment (*SLEEPS*; *n*_teacher_ = 4, *n*_student_ = 61) or control condition (standard curriculum not focusing on sleep; *n*_teacher_ = 4, *n*_student_ = 43). Multilevel‐ANCOVAs were conducted to examine the effects of *SLEEPS* on the theoretical mechanisms of sleep‐behaviour change (sleep‐related belief, attitudes, self‐efficacy, subjective social norms and intentions), sleep (*sleep behaviours* and quality) and related outcomes (internalising symptoms, academic motivation and engagement; See Section 2.3). The authors also explored whether the effects of *SLEEPS* varied across subgroups based on adolescents' demographics or baseline status. Outcomes were assessed at baseline and 8‐week post‐test (2 weeks after completing *SLEEPS*). Compared to the control group, adolescents receiving *SLEEPS* showed significantly larger improvement in their sleep‐related belief and self‐efficacy, *daytime sleepiness* and internalising symptoms. Also, students with less baseline belief about sleep hygiene improved more from *SLEEPS* than peers with more baseline belief.

**Trial Registration:** This trial was retrospectively registered at OSF Preregistration on 2/1/2025 [osf.io/9skf5]

## 1 Introduction

Like many countries globally, a significant proportion of middle school students in the US get less than the recommended 8–10 h of sleep every night (CDC 2020). This prevalent “sleep debt” can impair adolescents' cognitive, social–emotional and behavioural regulation, which is concerning given the multitude of social and academic demands on adolescents that require these skills (Mitchell et al. 2024). Conversely, adolescents who get adequate sleep consistently outperformed those who lack sufficient sleep across physical health (e.g., immune systems), mental health (e.g., subjective well‐being) and academic domains (e.g., *school engagement*, achievement; Gaskin et al. 2024). As myriad developmental domains and school‐related outcomes are impacted by adolescents' sleep, sleep has gained mounting traction in the field of school‐based intervention research worldwide (Rigney et al. 2021). The lack of access to feasible and theory‐informed sleep support in school could place adolescents at increased vulnerability for impairments in social–emotional, behavioural regulation and cognitive functioning that interfere with their academic performance. Hence, our multidisciplinary team (e.g., public health, medicine, education) developed a theory‐informed universal intervention program (Sleep to Enhance Educational Performance in Schools; *SLEEPS*) and piloted it in a cluster randomised trial (CRT) to test its effects on adolescents' sleep and related outcomes in middle schools.

### 1.1 *Sleep Behaviours* and Quality of Middle School Students

Sleep is a universal biological need that impacts adolescents' psychological, physiological and academic functioning (Mitchell et al. 2024). Two critical aspects of sleep are *sleep quality* and *sleep behaviours*. There are many ways to measure *sleep quality* (Fabbri et al. 2021). Specific to adolescents' school life and the scope of school‐based sleep interventions, one commonly used indicator of students' *sleep quality* is *daytime sleepiness*, which has been established in the literature to exert strong direct impact on students' daytime functioning at school and academic performance among common indicators of *sleep quality* (e.g., sleep duration, nocturnal awakening; Dewald et al. 2010). *Sleep quality* is the result of healthy *sleep behaviours*/*hygiene* (e.g., bedtime routine; Rigney et al. 2021).

Middle school students' sleep warrants special attention because early adolescents' biological changes in sleep are compounded by increasing environmental and social demands (e.g., social jetlag; Tonetti et al. 2022). For instance, early adolescents develop up to a 2‐h sleep–wake delay (later sleep onset and wake times) relative to middle childhood, while their optimal sleep duration remains around 8–10 h/night (Kohyama 2024). This circadian rhythm change is further complicated by their school start time, increased needs and demands for schoolwork and socialisation. These facts contribute to various unhealthy *sleep behaviours* prevalent among middle school students, such as excessive use of electronics beyond bedtime and irregular sleep–wake schedules (Hale and Dzierzewski 2024). Taken together, the prevalent unhealthy *sleep behaviours* put middle school students at heightened risk for inadequate *sleep quality*, which can significantly impair their school functioning (Mitchell et al. 2024).

### 1.2 School‐Based Universal Intervention Program for Sleep Hygiene

School represents an ideal setting for universal sleep programs because it offers students reliable and public‐funded access to on‐site professionals and evidence‐based interventions (Duong et al. 2021). School‐based sleep education and intervention programs have received mounting scholarly attention in the past decade (Pegado et al. 2023). However, researchers have identified the need for more theory‐informed programs for middle schoolers that go beyond sleep education to change *sleep behaviours* (Rigney et al. 2021). *SLEEPS* is a universal intervention program targeting middle school students' *sleep behaviours* to improve their sleep hygiene and academic enablers. *SLEEPS* was developed and refined in an iterative, co‐productive participatory procedure following the general procedure of the Intervention Mapping approach (Eldredge et al. 2016; See Data S1 for details of these qualitative studies). Building off the results from the co‐productive process with school stakeholders (e.g., teachers, students, caregivers, administrators) and experts, the authors integrated evidence‐based information and practices about adolescent sleep into six 30‐min classroom‐based interactive learning modules. Besides sleep‐related psychoeducation content, the authors embedded into *SLEEPS* three evidence‐based behaviour change strategies driven by social‐cognitive theories to promote students' translation of their learning into *sleep behaviour* change in life beyond schools (see Data S1 for details).

### 1.3 Theoretical Basis of *SLEEPS*

**FIGURE 1:**
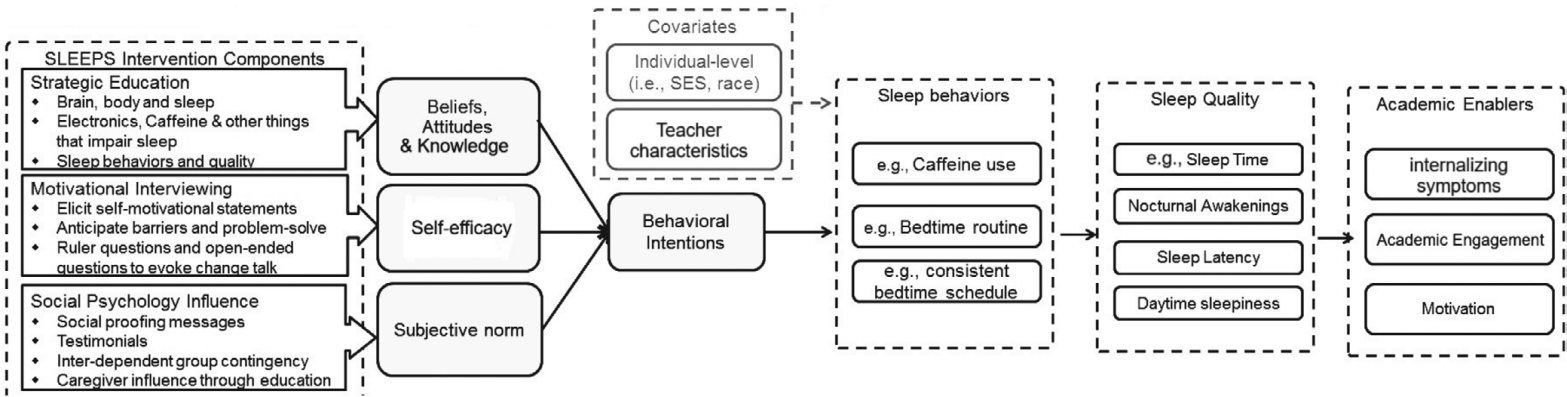
The intervention components, theoretical mechanisms of change and target outcomes of adolescent sleep and academic enablers.

The development and change mechanisms of *SLEEPS* (Figure 1) were grounded in the Theory of Planned Behaviour (TPB; Worthington 2021). Based on the TPB, the strongest predictor of adolescents' *sleep behaviour* change is their intention, which depends on three social‐cognitive factors: (a) whether one is in favour of healthy *sleep behaviours* (supportive attitudes), (b) how much one perceives social pressure from peers and adults to engage in healthy sleep (subjective norm) and (c) whether one believes in their control of healthy sleep (self‐efficacy). Specifically, *SLEEPS* was hypothesized to first improve adolescents' three social‐cognitive factors with psychoeducation and behaviour change strategies. Next, these improved factors would enhance adolescents' intentions and enactment of healthy *sleep behaviours* and academic enablers. Thus, the program component and outcome measures of *SLEEPS* (Figure 1) were deliberately designed to target the social‐cognitive factors based on TPB as the *mechanisms of change*. The three primary components of *SLEEPS* include (a) Strategic Education to foster adolescents' supportive beliefs and attitudes toward sleep hygiene, (b) Motivational Interviewing to enhance adolescents' self‐efficacy about sleep hygiene and (c) a set of Social Influence Activities to promote adolescents' subjective norms about sleep hygiene (Supplemental file S1).

### 1.4 Gaps in Literature and Study Objectives

While school‐based universal sleep programs have increased over the last decade, there are several limitations to address (Gaskin et al. 2024). First, the field needs more school‐based sleep programs rooted in the change mechanisms derived from established theories of behavioural change (Busch et al. 2017). A theory‐informed change mechanism is crucial for effective program development because it explains adolescents' behaviour change process in terms of social, cognitive and behavioural factors that are amenable to behaviour change strategies in schools (e.g., TPB; Inhulsen et al. 2022). Second, compared to primary and high school students, fewer school‐based sleep programs were designed to developmentally target middle school students (Gaskin et al. 2024). Most middle school students start or have started entering early adolescence, during which many neurodevelopmental changes in sleep occur (e.g., delayed circadian rhythm), providing the impetus for sleep programs specifically developed for middle school. Third, many existing sleep programs for adolescents have been developed by researchers or clinicians in the academia or clinical context, not by school stakeholders in the authentic school setting (Rigney et al. 2021), which misses the microsystems that constantly influence adolescent sleep (e.g., peer social norms).

To narrow these gaps, the authors conducted this trial to explore the preliminary effectiveness of *SLEEPS* on middle school students' sleep‐related outcomes. Three sequential research questions (RQs) guided this study. RQ1: Compared to the control group, did students receiving *SLEEPS* improve more in their TPB change mechanisms and sleep‐related outcomes while controlling for student demographics and baseline outcomes? RQ2: For the outcomes identified as significant in RQ1, did student baseline outcomes moderate the effect of *SLEEPS* on the same outcomes at post‐test? RQ3: For the outcomes identified as significant in RQ1, did student demographics moderate the effect of *SLEEPS* on students' outcomes at post‐test?

## 2 Methods

### 2.1 Setting and Participants

Participants (teachers and students in their classes) were recruited from eight middle schools in a large metropolitan area in the Midwest US At the time of this study, the participating schools served a diverse student enrollment. This school‐level CRT generated a nested data structure wherein 104 participating students were nested in eight teachers' classes. Teachers were mostly female (75%), White (100%) and had less than 10 years of work experience (Table 1). Students were 11–14‐year‐old (*M* = 12.4), in sixth to eighth grade, 54.37% female, 89.69% White and 44.23% receiving FRPL (Table 1).

**TABLE 1:** Descriptive statistics of participants and baseline equivalences (*n*_teacher_ = 8, *n*_student_ = 104).

Categorical variables	Categories	*n*	Percentage	Baseline equivalence between study conditions
School/teacher: Level 2				
Condition	Control	4	50	—
	Treatment	4	50	
Gender	Male	2	25	*χ*2 (1, 8) = 2.67, *p* = 0.10
	Female	6	75	
Education level	Bachelor	2	25	*χ*2 (1, 8) = 0, *p* = 1
	Master's degree	6	75	
Teaching experience (years)	< 8	3	37.50	*χ*2 (2, 8) = 2.67, *p* = 0.26
	8–9.25	3	37.50	
	9.25+	2	25	
Student: Level 1				
Condition	Control	43	41.35	—
	Treatment	61	58.65	
Gender	Male	47	45.63	*χ*2 (1, 104) = 5.09, *p* = 0.02*
	Female	56	54.37	
Race/ethnicity	White	87	89.69	*χ*2 (6, 104) = 6.14, *p* = 0.41
	Asian/Asian American	2	2.06	
	Black/African American	3	3.09	
	Hispanic/Latino	1	1.03	
	Native Hawaiian/Pacific Islander	1	1.03	
	American Indian or Alaska Native	1	1.03	
	Other	2	2.06	
FRPL	No	58	55.77	*χ*2 (1, 104) = 0.54, *p* = 1
	Yes	46	44.23	
Age (year‐old)	11	21	20.19	*χ*2 (3, 104) = 2.46, *p* = 0.12
	12	36	34.62	
	13	21	20.19	
	14	26	25	

*Note*: The demographics were coded as categorical variables, which were entered into the ML‐ANCOVA models as *n* − 1 dummy variables (*n* is the number of categories). “—” = not applicable.

Abbreviation: FRPL = free/reduced price lunch.

* *p* < 0.05.

### 2.2 Procedure

**FIGURE 2:**
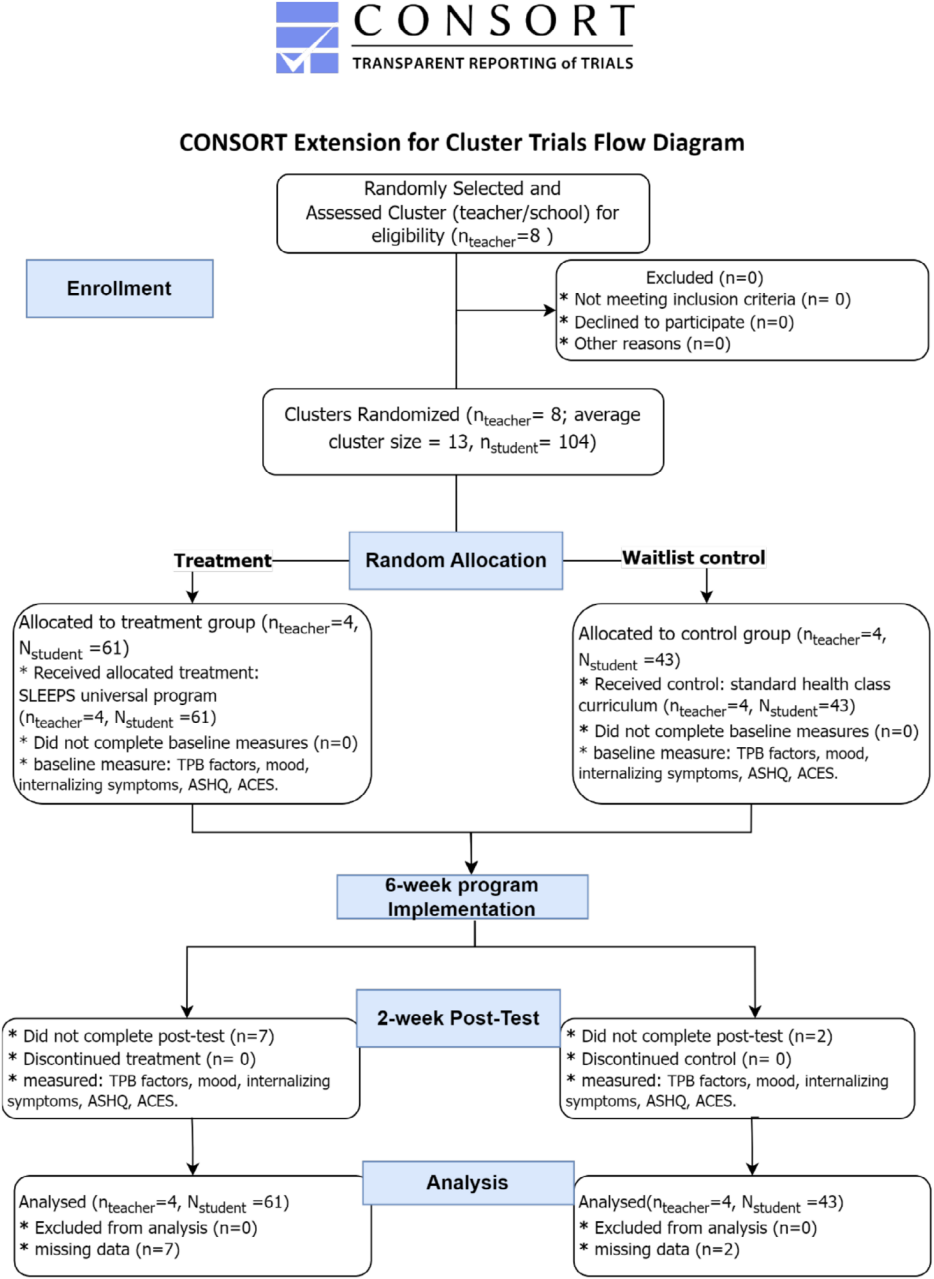
The CONSORT cluster randomised trial extension diagram delineates the design, timeline of enrollment, randomization, pre‐test, treatment implementation and data collection. *Note*: TPB factors include beliefs, attitudes, self‐efficacy, subjective norm and intention about engaging healthy *sleep behaviours*. ACES = Academic Competence Evaluation Scales, ASHQ = Adolescent Sleep Habit Questionnaire.

This cluster‐randomised trial was approved by the authors' University IRB. In Spring 2021, the authors recruited and obtained informed consent from one teacher in each of the eight middle schools. Then, the authors collaborated with the participating teachers to recruit and obtain informed consent from the legal guardians of the students in their classes. To reduce baseline variance between study groups, after consenting, the eight teachers and their corresponding classes were paired based on their school demographics via the nearest‐neighbour matching approach (Dang et al. 2021). Each teacher/class in a pair was then randomly allocated to either the Control (*n* = 43, “business as usual” with existing standard curriculum) or Treatment Group (*n* = 61, *SLEEPS*). The random allocation was single‐masked against the data analyst. To facilitate causal inference, the authors separated the study components sequentially across 2 months as depicted in the CONSORT diagram (Figure 2). The authors followed the CONSORT checklist in trial reporting (Data S2). Participants in both study conditions were assigned a random ID to ensure anonymity and enable the match of their pre‐and post‐test responses to anonymous online surveys (see Section 2.3). To ensure response rates, the authors provided participants with weekly reminders and incentives. The attrition rate from pre‐to post‐test was 8.65% for students and 0% for teachers in the final sample.

#### 2.2.1 Treatment Group: *SLEEPS*

Teachers in the treatment group completed a 2‐ to 3‐h online training about *SLEEPS* due to COVID‐19 confinement (See Data S1 for details). Throughout the 6‐week implementation phase, each of the four teachers in the treatment group delivered the *SLEEPS* curriculum to their corresponding class of students in an online or hybrid format (depending on school‐specific pandemic mandates). Teachers taught each of the six modules in one or two class sessions following both “synchronous” (i.e., teacher‐led live lessons) and “asynchronous” modalities (i.e., independent learning beyond class). Based on the suggestions of stakeholders and to support teachers' implementation fidelity, the authors created a web platform (see Data S1) to offer participants various online resources, such as pre‐recorded videos to aid in‐class instruction and sleep‐related enrichment materials.

#### 2.2.2 Control Group

Teachers in the control group continued implementing their existing standard academic curriculum (focusing on sleep‐irrelevant subjects, e.g., math) for the same 6‐week duration as *SLEEPS*. Upon study completion, the control group teachers received training and implemented *SLEEPS* for the control group students.

### 2.3 Measures

#### 2.3.1 Beliefs About Sleep

The authors designed a 4‐item scale to assess adolescents' meta‐cognitive perceptions and beliefs about what, why and how to engage in healthy sleep (Data S3). We measured perceptions and beliefs about sleep, instead of specific sleep knowledge, because it is the immediate pre‐determinant to attitude changes based on TPB (Worthington 2021). Also, the items were designed based on the learning objectives of *SLEEPS*. Each item was rated by adolescents on a 4‐point Likert scale ranging from 1 = “*Not at all true*” to 4 = “*Very true*”. A higher total score indicates an adolescent holds more supportive beliefs about the importance of sleep. In this sample, it demonstrated adequate internal consistency (McDonald's *ω* = 0.78). The authors computed McDonald's *ω* instead of Chronbach's *α* for the small‐sized scales in this study because *ω* outperforms *α* for scales consisting of small numbers of items (Dunn et al. 2014).

#### 2.3.2 TPB Change Mechanisms Factors

Each of the four TPB factors was assessed via a three‐item self‐report measure based on empirical assessment guidelines of TPB constructs (Francis et al. 2004; Data S3). The Attitude Scale captures adolescents' attitudes toward engaging in healthy *sleep behaviours*. The Subjective Norms Scale captures adolescents' perceived peer and adult social norms about healthy *sleep behaviours*. The Self‐Efficacy Scale captures adolescents' self‐efficacy related to engaging in healthy *sleep behaviours*. The Behavioural Intention Scale assesses adolescents' intentions to engage in healthy *sleep behaviours*. Items were rated by students on a five‐point Likert scale ranging from 1 = “*Not at all true*” to 5 = “*Completely true*.” A higher score indicates a positive TPB factor. In this sample, the Attitude, Self‐Efficacy, Subjective Norms and Intention Scales consistently showed acceptable reliabilities (*ω* = 0.75, 0.85, 0.72, 0.87, respectively).

#### 2.3.3 Sleep Behaviours

To comprehensively assess the aspects of sleep hygiene specifically targeted by *SLEEPS*, we selected three scales from established measures of adolescents' sleep hygiene (e.g., Adolescent Sleep Hygiene Scale‐revised, Storfer‐Isser et al. 2013; School Sleep Habits Survey, Owens et al. 2010; Wolfson et al. 2003). This resulted in a combined self‐report measure of 24 items to be rated by adolescents on a five‐point Likert scale ranging from 0 “*Never: 0 times in the past 2 weeks*” to 4 “*Almost always: 5 or more times per week*” based on their sleep–wake behaviours in the past 2 weeks. It yields three subscale scores (*Sleep Routine and Environment*, *Technology Use During Bedtime*, *Sleep‐Related Cognition and Emotion*), while the total score captures overall sleep hygiene. To enhance score interpretability, the items were reverse‐coded so that higher scores indicate healthier sleep hygiene. In this sample, *Sleep Routine, Technology Use, Sleep‐Related Cognition and Emotion* and total scale demonstrated acceptable reliabilities (*ω* = 0.67, 0.69, 0.62, 0.79, respectively).

#### 2.3.4 Daytime Sleepiness

Based on inputs from school stakeholders and the scope of *SLEEPS* as a school‐based sleep program, we measured adolescents' *daytime sleepiness* at school as the school‐focused indicator of their *sleep quality* via the Paediatric Daytime Sleepiness Scale (PDSS; Drake et al. 2003). Adolescents rated the eight items in this scale on a 5‐point Likert scale ranging from 0 = “*Never in the past two weeks*” to 4 = “*five or more times per week*” based on some key indicators of *sleep quality*, *daytime sleepiness* and functioning at home and school. To enhance result interpretability, the items were reverse‐coded so that higher scores represent better *sleep quality*. In this sample, the PDSS showed acceptable reliability (*ω* = 0.74).

#### 2.3.5 Internalising Symptoms

Adolescents' internalising symptoms were assessed via the internalising symptom subscale of the Teen Sleep Habits Survey (Wahlstrom et al. 2014). This five‐item subscale was rated by adolescents on a three‐point Likert scale ranging from 0 = “*Never*” to 2= “*Always*” based on their experience in the past 2 weeks. A higher total mean score indicates that an adolescent more frequently experiences internalising symptoms recently. In this sample, it demonstrated good reliability (*ω* = 0.81).

#### 2.3.6 Academic Enablers

Adolescents' academic enablers were measured with the Academic Competence Evaluation Scales (ACES, DiPerna 2005). To ensure the feasibility and focus of this study, the authors used the five‐item *Academic Motivation* and nine‐item *School engagement* subscales of the ACES that were closely related to adolescents' sleep. The items were rated by adolescents on a five‐point Likert scale ranging from 1 “*Never*” to 5 “*Almost always*”. A higher score indicates stronger motivation or engagement. In this sample, the subscales demonstrated adequate reliabilities (*Motivation: ω* = 0.87, *Engagement: ω* = 0.79).

#### 2.3.7 Demographics

We controlled for participants' demographics as covariates in the ML‐ANCVOA, including teachers' experience, students' age, gender, race and FRPL status (Table 1).

#### 2.3.8 *SLEEPS* Intervention Fidelity

The authors embedded in *SLEEPS* an intervention fidelity measure to assess the extent to which participating teachers properly delivered the core components of *SLEEPS* following guidelines from Sanetti and Kratochwill (2011; Data S3). This pragmatic measure was developed based on input from expert panels and school stakeholders to enable teachers to feasibly self‐report four subscales/dimensions of intervention fidelity. Based on schools' requirements, the self‐report method was deemed the most acceptable and feasible way to assess fidelity during COVID‐19 confinement. To enhance the validity, the authors built the items based on observable facts that limit educators' subjectivity and self‐serving bias in self‐reporting (Sanetti and Kratochwill 2011).

### 2.4 Data Analysis

First, independent‐sample *t*‐tests (baseline outcomes) and Chi‐square tests (demographics) were run to confirm the baseline equivalences between study conditions (Table 2). Next, bivariate correlation matrixes were calculated among all variables (Data S4). For intra‐class correlations (ICC), unconditional models were fitted by regressing only the random level 1 intercept on the 12 sleep‐related outcomes (change mechanisms, *sleep behaviours*, *daytime sleepiness*, internalising symptoms and academic enablers). The descriptive statistics (e.g., normal residual distribution, ICCs; Table 3) supported the sample adequacy for ML‐ANCOVAs (Osborne and Waters 2002).

**TABLE 2:** Independent samples *t*‐tests of pre‐tests of outcomes between treatment and control conditions.

Baseline outcomes		Mean		Standard deviation		*t*‐test for equality of means		
		Ctrl	Trt	Ctrl	Trt	*t*	df	*p*
Adolescent *daytime sleepiness*		2.59	2.88	0.63	0.43	2.75	102	0.01*
Adolescent sleep hygiene	*Sleep routine and environment*	2.50	2.69	0.50	0.51	1.89	102	0.06
	Sleep‐related mood	1.91	2.10	0.56	0.48	1.81	102	0.07
	Electronic use	1.22	1.32	0.44	0.46	1.03	102	0.31
Mechanisms of change based on the Theory of Planned Behaviours	Sleep beliefs	3.05	3.28	0.66	0.47	2.14	102	0.03*
	Attitudes	3.90	3.85	0.81	0.78	−0.30	102	0.77
	Self‐efficacy	3.32	3.79	1.05	0.77	2.66	102	0.01*
	Subjective norms	3.29	3.57	0.55	0.66	2.22	102	0.03*
	Behavioural intentions	3.52	3.82	0.91	0.77	1.82	102	0.07
Internalising symptoms		0.73	0.54	0.53	0.36	−2.20	102	0.03*
Academic enablers	*School engagement*	3.73	3.93	0.79	0.67	1.45	102	0.15
	Academic motivation	3.67	3.86	0.71	0.66	1.37	102	0.17

Abbreviations: Ctrl = Waitlist control; Trt = *SLEEPS*.

* *p* < 0.05.

**TABLE 3:** Intra‐class correlations (ICCs) and variance components of the random intercept from the unconditional models.

Outcome variables	Random effects	Variance component	df	Chi‐square	*p*	ICC	SMDES
Adolescent *daytime sleepiness*	Level 1 intercept ($\mu_{0j}$)	0.06	7	27.32	< 0.001	0.19***	0.22
	Level 1 residual ($r_{\mathrm{ij}}$)	0.25					
*Sleep routine and environment*	Level 1 intercept ($\mu_{0j}$)	0.04	7	24.67	< 0.001	0.18***	0.01
	Level 1 residual ($r_{\mathrm{ij}}$)	0.21					
Sleep‐related mood	Level 1 intercept ($\mu_{0j}$)	0.05	7	20.05	< 0.05	0.14*	0.07
	Level 1 residual ($r_{\mathrm{ij}}$)	0.33					
Electronic use	Level 1 intercept ($\mu_{0j}$)	0.05	7	26.48	< 0.001	0.19***	−0.04
	Level 1 residual ($r_{\mathrm{ij}}$)	0.20					
Sleep beliefs	Level 1 intercept ($\mu_{0j}$)	0.03	7	17.36	< 0.05	0.11*	0.16
	Level 1 residual ($r_{\mathrm{ij}}$)	0.26					
Attitudes	Level 1 intercept ($\mu_{0j}$)	0.06	7	11.94	0.10	0.06	−0.09
	Level 1 residual ($r_{\mathrm{ij}}$)	0.95					
Self‐efficacy	Level 1 intercept ($\mu_{0j}$)	0.04	7	11.12	0.13	0.05	0.07
	Level 1 residual ($r_{\mathrm{ij}}$)	0.77					
Subjective norms	Level 1 intercept ($\mu_{0j}$)	0.01	7	9.44	0.22	0.04	−0.24
	Level 1 residual ($r_{\mathrm{ij}}$)	0.33					
Behavioural intentions	Level 1 intercept ($\mu_{0j}$)	0.15	7	30.47	< 0.001	0.19***	−0.1
	Level 1 residual ($r_{\mathrm{ij}}$)	0.62					
Internalising symptoms	Level 1 intercept ($\mu_{0j}$)	0.08	7	21.03	< 0.001	0.14***	−0.09
	Level 1 residual ($r_{\mathrm{ij}}$)	0.48					
*School engagement*	Level 1 intercept ($\mu_{0j}$)	0.13	7	35.27	< 0.001	0.23***	−0.06
	Level 1 residual ($r_{\mathrm{ij}}$)	0.44					
Academic motivation	Level 1 intercept ($\mu_{0j}$)	0.01	7	8.88	0.26	0.03	−0.03
	Level 1 residual ($r_{\mathrm{ij}}$)	0.18					

*Note*: Level 1: *N* = 104; Level 2: *N* = 8.

* *p* < 0.05.

*** *p* < 0.001.

For the three RQs, a series of two‐level ML‐ANCOVAs were configured (see Data S5 for equations). The ML‐ANCOVAs enabled us to account for the nested data (students are nested in teachers/classes) and interactions between class‐level treatment and individual‐level covariates. For RQ1, the main effect models were fitted for each outcome (Table 4). In the level 1 (individual‐level) equations, the post‐test of an outcome was entered as the dependent variable while controlling for the baseline outcome and demographics. The random level 1 intercept was set to vary across teachers/classes and was predicted by study condition (*SLEEPS* vs. Control) and teacher demographics in the level 2 (class‐level) equations. For RQs 2 and 3, the models of RQ1 were extended by adding study condition to the level 2 equations of the slopes of baseline outcomes or demographics to explore if the treatment effect of *SLEEPS* varied across students with different baseline statuses or demographic groups (Tables 4 and 5). Based on the hierarchy rule of moderation analysis, interaction models were only fitted with baseline outcomes identified as significant in RQ1 (Hayes and Rockwood 2020). For significant interactions, class‐specific regression lines were plotted between the pre‐ and post‐tests values and grouped by two smoothed regression lines representing the treatment versus control conditions.

**TABLE 4:** Fixed effect estimates of ML‐ANCOVAs controlling for baseline outcome and demographics.

Outcome variables	Statistics	Level 2: Class/teacher level			Level 1: Student level					
		Intercept	Treatment	Teaching experience	Age	Race	Gender	FRPL	Baseline status	Baseline × treatment
Adolescent *daytime sleepiness*	*b* (SE)	**3.823 (0.556)**	**0.356 (0.106)**	0.001 (0.001)	**−0.095 (0.044)**	−0.014 (0.114)	−0.068 (0.073)	−0.007 (0.048)	**0.732 (0.07)**	—
	Partial Cohen's *d*	6.153	3.008	0.857	−0.484	−0.027	−0.210	−0.034	2.346	—
	*p*	< 0.001	0.025	0.383	0.034	0.904	0.355	0.882	< 0.001	—
*Sleep routine and environment*	*b* (SE)	**4.417 (0.587)**	0.222 (0.121)	−0.002 (0.002)	**−0.159 (0.047)**	0.052 (0.117)	0.103 (0.069)	−0.036 (0.046)	**0.61 (0.075)**	—
	Partial Cohen's *d*	6.734	1.639	−0.902	−0.766	0.100	0.337	−0.176	1.840	—
	*p*	< 0.001	0.125	0.360	0.001	0.658	0.138	0.437	< 0.001	—
Sleep‐related mood	*b* (SE)	**3.669 (0.61)**	0.275 (0.117)	0.002 (0.002)	**−0.141 (0.049)**	−0.188 (0.124)	−0.098 (0.078)	−0.009 (0.052)	**0.619 (0.079)**	—
	Partial Cohen's *d*	5.384	2.093	0.863	−0.649	−0.339	−0.284	−0.041	1.763	—
	*p*	< 0.001	0.065	0.379	0.006	0.136	0.211	0.857	< 0.001	—
Electronic use	*b* (SE)	**2.184 (0.632)**	−0.027 (0.178)	−0.003 (0.003)	−0.072 (0.05)	**−0.253 (0.105)**	−0.054 (0.063)	0.011 (0.043)	**0.74 (0.081)**	—
	Partial Cohen's *d*	3.088	−0.138	−0.970	−0.326	−0.543	−0.192	0.057	2.064	—
	*p*	0.024	0.884	0.328	0.151	0.018	0.395	0.802	< 0.001	—
Main effect model: Sleep beliefs	*b* (SE)	**3.963 (0.452)**	**0.454 (0.076)**	−0.002 (0.001)	**−0.076 (0.036)**	0.131 (0.122)	0.134 (0.078)	−0.016 (0.052)	**0.659 (0.069)**	—
	Partial Cohen's *d*	7.839	5.315	−1.665	−0.469	0.241	0.387	−0.070	2.135	—
	*p*	< 0.001	< 0.001	0.120	0.040	0.287	0.089	0.756	< 0.001	—
Baseline interaction model: sleep beliefs	*b* (SE)	**4.106 (0.443)**	**0.452 (0.074)**	−0.002 (0.001)	**−0.085 (0.035)**	0.146 (0.118)	0.106 (0.076)	−0.023 (0.051)	**0.778 (0.084)**	−0.332 (0.137)
	Partial Cohen's *d*	8.292	5.457	−1.720	−0.546	0.278	0.315	−0.105	2.108	−0.546
	*p*	< 0.001	< 0.001	0.111	0.018	0.223	0.169	0.645	< 0.001	0.018
Attitudes	b (SE)	**4.682 (1.145)**	−0.011 (0.195)	**−0.007 (0.003)**	−0.066 (0.092)	−0.353 (0.289)	0.107 (0.19)	0.104 (0.122)	**0.74 (0.128)**	—
	Partial Cohen's *d*	3.657	−0.049	−2.338	−0.161	−0.274	0.127	0.193	1.304	—
	*p*	0.013	0.959	0.047	0.476	0.227	0.574	0.394	< 0.001	—
Self‐efficacy	*b* (SE)	**4.339 (0.993)**	**0.544 (0.178)**	−0.004 (0.002)	−0.08 (0.079)	0.177 (0.223)	−0.146 (0.142)	0.085 (0.095)	**0.622 (0.079)**	—
	Partial Cohen's *d*	3.909	2.737	−1.261	−0.227	0.179	−0.231	0.201	1.772	—
	*p*	0.008	0.031	0.218	0.317	0.429	0.309	0.376	< 0.001	—
Subjective norms	*b* (SE)	**4.151 (0.8)**	0.236 (0.146)	0 (0.002)	−0.077 (0.064)	0.086 (0.176)	−0.136 (0.111)	0.072 (0.074)	**0.425 (0.1)**	—
	Partial Cohen's *d*	4.641	1.438	−0.086	−0.270	0.110	−0.277	0.220	0.957	—
	*p*	0.001	0.168	0.928	0.233	0.626	0.223	0.332	< 0.001	—
Behavioural intentions	*b* (SE)	**4.98 (1.274)**	0.164 (0.299)	−0.005 (0.004)	−0.108 (0.101)	**−0.561 (0.22)**	0.065 (0.135)	0.003 (0.091)	**0.618 (0.09)**	—
	Partial Cohen's *d*	3.497	0.490	−0.998	−0.241	−0.575	0.108	0.008	1.540	—
	*p*	0.016	0.607	0.316	0.288	0.013	0.631	0.973	< 0.001	—
*School engagement*	*b* (SE)	**4.229 (1.003)**	0.174 (0.211)	−0.006 (0.003)	−0.037 (0.08)	0.05 (0.191)	−0.06 (0.122)	−0.117 (0.081)	**0.573 (0.089)**	—
	Partial Cohen's *d*	3.770	0.741	−1.849	−0.104	0.059	−0.111	−0.324	1.449	—
	*p*	0.011	0.445	0.092	0.644	0.792	0.623	0.154	< 0.001	—
Academic motivation	*b* (SE)	**4.397 (0.975)**	0.214 (0.208)	−0.007 (0.003)	−0.061 (0.078)	−0.219 (0.179)	0.056 (0.112)	−0.087 (0.076)	**0.635 (0.084)**	—
	Partial Cohen's *d*	4.035	0.924	−2.040	−0.178	−0.276	0.114	−0.257	1.699	—
	*p*	0.006	0.349	0.070	0.432	0.225	0.615	0.258	< 0.001	—
Internalising symptoms	*b* (SE)	0.084 (0.381)	**−0.18 (0.065)**	−0.001 (0.001)	0.044 (0.031)	−0.163 (0.104)	**0.155 (0.066)**	**0.088 (0.043)**	**0.681 (0.075)**	—
	Partial Cohen's *d*	0.199	−2.486	−1.175	0.322	−0.350	0.528	0.456	2.056	—
	*p*	0.834	0.039	0.246	0.156	0.123	0.022	0.046	< 0.001	—

*Note*: For easy navigation, coefficients with significant *p* values were bolded. “—” indicates not applicable. The left column of “Baseline × Treatment” contains relevant results from the interaction models (RQ2), while the other columns contains results from the main effect models (RQ1).

Abbreviations: *b* = unstandardized fixed effect coefficient; SE = standard error; treatment = *SLEEPS* versus business‐as‐usual.

**TABLE 5:** Fixed effect estimates of ML‐ANCOVAs for the interactions between student/teacher demographics and treatment.

		Level 2: Class/teacher level				Level 1: Student level								
		Intercept	Treatment	Teaching exp	Exp × Trt	Age	Age × Trt	Race	Race × Trt	Gender	Gender × Trt	FRPL	FRPL × Trt	Baseline
Adolescent *daytime sleepiness*	*b* (SE)	2.827 (1.007)	1.84 (1.301)	0.002 (0.002)	—	−0.015 (0.081)	−0.12 (0.104)	0.011 (0.116)	—	−0.076 (0.073)	—	0.003 (0.049)	—	0.731 (0.07)
	Partial Cohen's *d*	2.510	1.265	1.146		−0.042	−0.259	0.021		−0.235		0.015		2.366
	*p*	0.038	0.216	0.257		0.855	0.256	0.926		0.302		0.948		< 0.001
*Sleep routine and environment*	*b* (SE)	5.011 (1.037)	−0.692 (1.329)	−0.002 (0.002)	—	−0.207 (0.083)	0.074 (0.106)	0.036 (0.119)	—	0.106 (0.069)	—	−0.04 (0.047)	—	0.609 (0.075)
	Partial Cohen's *d*	4.323	−0.466	−0.994		−0.562	0.156	0.069		0.348		−0.196		1.847
	*p*	0.003	0.624	0.318		0.015	0.491	0.760		0.128		0.391		< 0.001
Sleep‐related mood	*b* (SE)	3.757 (1.114)	0.135 (1.433)	0.002 (0.002)	—	−0.148 (0.09)	0.011 (0.115)	−0.191 (0.127)	—	−0.099 (0.078)	—	−0.01 (0.053)	—	0.618 (0.079)
	Partial Cohen's *d*	3.017	0.084	0.735		−0.373	0.022	−0.339		−0.284		−0.041		1.763
	*p*	0.025	0.929	0.448		0.103	0.923	0.138		0.213		0.859		< 0.001
Electronic use	*b* (SE)	2.142 (0.643)	−0.017 (0.18)	−0.003 (0.003)	—	−0.069 (0.051)	—	−0.211 (0.142)	−0.091 (0.209)	−0.05 (0.064)	—	0.008 (0.043)	—	0.742 (0.081)
	Partial Cohen's *d*	2.982	−0.086	−0.944		−0.311		−0.337	−0.099	−0.178		0.043		2.069
	*p*	0.026	0.928	0.340		0.174		0.140	0.663	0.434		0.849		< 0.001
Sleep beliefs	*b* (SE)	4.765 (0.972)	−0.864 (1.25)	−0.003 (0.001)	—	−0.141 (0.079)	0.106 (0.101)	0.108 (0.124)	—	0.142 (0.078)	—	−0.026 (0.053)	—	0.665 (0.069)
	Partial Cohen's *d*	4.386	−0.618	−1.761		−0.405	0.239	0.196		0.413		−0.113		2.170
	*p*	0.003	0.520	0.105		0.077	0.295	0.390		0.072		0.620		< 0.001
Attitudes	*b* (SE)	3.57 (1.455)	−0.074 (0.192)	−0.005 (0.003)	−0.009 (0.008)	0.024 (0.117)	—	−0.286 (0.296)	—	0.113 (0.19)	—	0.104 (0.122)	—	0.753 (0.128)
	Partial Cohen's *d*	2.454	−0.387	−1.703	−1.175	0.047		−0.219		0.135		0.194		1.329
	*p*	0.067	0.718	0.163	0.306	0.838		0.337		0.552		0.395		< 0.001
Behavioural intentions	*b* (SE)	4.976 (1.293)	0.167 (0.304)	−0.005 (0.004)	—	−0.108 (0.102)	—	−0.549 (0.299)	−0.028 (0.446)	0.066 (0.137)	—	0.002 (0.093)	—	0.618 (0.091)
	Partial Cohen's *d*	3.441	0.492	−0.991		−0.239		−0.416	−0.014	0.109		0.005		1.539
	*p*	0.017	0.605	0.319		0.295		0.069	0.951	0.631		0.981		< 0.001
Academic motivation	*b* (SE)	0.94 (1.094)	0.003 (0.15)	−0.002 (0.002)	−0.007 (0.006)	0.028 (0.079)	—	−0.19 (0.18)	—	0.046 (0.111)	—	−0.082 (0.075)	—	0.656 (0.084)
	Partial Cohen's *d*	0.859	0.019	−1.044	−1.204	0.080		−0.239		0.094		−0.247		1.770
	*p*	0.439	0.986	0.356	0.295	0.726		0.295		0.679		0.280		< 0.001
Internalising symptoms	*b* (SE)	0.218 (0.412)	−0.137 (0.128)	−0.001 (0.001)	—	0.03 (0.032)	—	−0.144 (0.105)	—	0.24 (0.109)	−0.146 (0.137)	0.051 (0.068)	0.075 (0.09)	0.684 (0.075)
	Partial Cohen's *d*	0.473	−0.960	−1.036		0.212		−0.312		0.502	−0.244	0.170	0.189	2.088
	*p*	0.619	0.333	0.299		0.355		0.176		0.031	0.288	0.457	0.410	< 0.001

*Note*: “—” indicates not applicable. “*x*” indicates the multiplicative interaction term between two variables.

Abbreviations: *b* = unstandardized fixed effect coefficient; Exp = experience; SE = standard error; Treatment/Trt = *SLEEPS* versus business‐as‐usual.

For all analyses, listwise deletion was used to handle missingness to obtain conservative estimates (missing rate < 0.05% only in demographics; Baraldi and Enders 2010). Power analysis was performed for the primary main effect models (RQ1) with Optimal Design 3.01. Given the sample sizes, seven covariates, an Alpha level of 0.05, an average ICC of 0.02 in post‐test outcomes, and 70% variance explained by level 2 covariates, we would have sufficient power (≥ 80%) to detect a minimum detectable effect size of 0.63. Two types of effect sizes were calculated (Brysbaert and Debeer 2025), including (a) standardised effect size of partial Cohen's *d* to compare across models within this study, and (b) *standardised mean differences effect size* (SMDES) to compare our findings to other studies'. The SMDES represents the number of pooled pre‐test standard deviations of the mean difference of pre‐ versus post‐tests between study conditions. Analyses were performed with HLM ver. 6.08 and SPSS ver. 26.

## 3 Results

The results of *χ* ^2^ tests and independent‐sample *t*‐tests indicated that the random allocation resulted in probabilistic equivalence between study conditions for most of the baseline outcomes and demographics (Tables 1 and 2). To adjust for the inequivalence identified in some key variables, baseline outcomes and demographics were controlled in the ML‐ANCOVAs (Data S5). The results of fidelity indicated that most participating teachers (a) adhered to the lesson curriculum (*M* = 3.71, SD = 0.46, range:3–4, maximum = 4), (b) reached the instructional time length recommended in the *SLEEPS* curriculum (30‐min, range: 20–55 min) and (c) properly engaged students (*M* = 3.25, SD = 0.53, range:2–4, maximum = 4). The correlation matrix revealed weak to moderate associations between study conditions and the post‐tests of most outcomes. Additionally, there were moderate associations among student age, FRPL status and the post‐tests of most outcomes (Data S4). The significant ICCs indicated that students from the same class share similarity in their sleep‐related outcomes at post‐test (Table 3), which warranted the appropriateness of using CRT and ML‐ANCOVA for this study.

### 3.1 RQ1: Main Effect of *SLEEPS*

The results (Tables 3 and 4) indicated that, compared to students in the control, students receiving *SLEEPS* reported significant improvement from pre‐test to post‐test in their *sleep‐related beliefs* (*b* = 0.45, *p* < 0.01; Partial Cohen's *d* = 5.32; SMDES = 0.16) and *self‐efficacy* about engaging in healthy *sleep behaviours* (*b* = 0.54, *p* < 0.05; Partial Cohen's *d* = 2.74; SMDES = 0.07), *daytime sleepiness* (*b* = 0.36, *p* < 0.05; Partial Cohen's *d* = 3; SMDES = 0.22) and *internalising symptoms* (*b* = −0.18, *p* < 0.05; Partial Cohen's *d* = −2.49; SMDES = ‐0.03). In other words, after adjusting for the influences of baseline status and demographics, students receiving *SLEEPS* experienced increased positive beliefs and self‐efficacy about engaging in healthy sleep, as well as decreased *daytime sleepiness* at school and negative moods (e.g., sadness). Conversely, compared to students in the control, students receiving *SLEEPS* reported no significant improvement from pre‐test to post‐test in their *attitudes, subjective norm, behavioural intention, sleep behaviours, academic motivation* or *school engagement*.

### 3.2 RQs 2 and 3: Interaction Between Treatment and Baseline Outcome/Demographics

**FIGURE 3:**
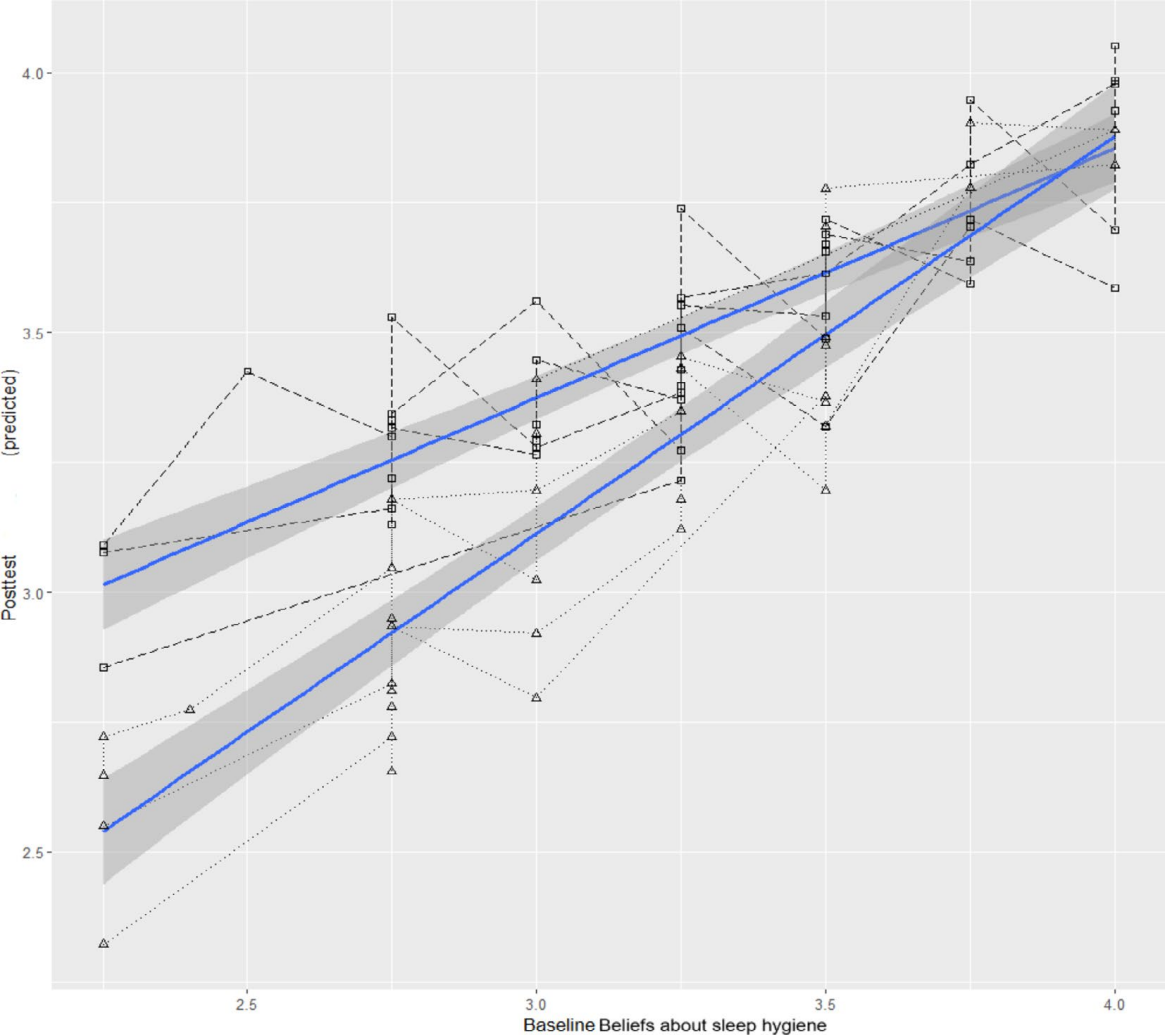
The significant cross‐level interaction effect between study conditions and baseline value on the post‐test value of beliefs about sleep hygiene. *Note*: Black lines represent class‐specific associations between baseline and post‐test beliefs about sleep hygiene, long‐dash lines with square dots = classes in treatment group, dotted lines with triangle dots = classes in control group. Blue solid lines = the smoothed linear regression lines fitted separately for each study condition (upper line = treatment group, lower line = control group), grey shades surrounding the solid lines = 95% confidence region. The axis was truncated (i.e., not starting from 0) to save space and highlight the range of baseline and post‐test scores in the current sample.

A significant cross‐level interaction was found between treatment and adolescents' baseline beliefs about healthy sleep (Table 4). For adolescents with strong baseline beliefs about healthy sleep, the post‐test beliefs of adolescents from treatment or control groups were similar as shown by the converging regression lines in the high baseline quadrant (upper right corner of Figure 3). Conversely, for adolescents struggling with weak baseline beliefs, the post‐test values of adolescents in the treatment group exceeded those of peers in the control group evidenced by the gap between lines in the low baseline quadrant (bottom left corner of Figure 3). Overall, the interaction effect implied that *SLEEPS* had a larger effect on adolescents holding weaker beliefs about sleep at baseline than peers holding stronger beliefs at baseline. Unlike RQ2, the models of RQ3 (Table 5) revealed no significant interactions between treatment and adolescents' demographics.

## 4 Discussion

There is a pressing need for theory‐informed universal intervention programs that target the sleep of middle school students to facilitate their social–emotional and behavioural development for success in school and life (Rigney et al. 2021). This study aimed to evaluate the effectiveness of *SLEEPS* developed by a multidisciplinary team on adolescents' sleep hygiene and sleep‐related outcomes in middle schools in the US. The findings preliminarily supported the positive effects of *SLEEPS* on improving adolescents' sleep‐related beliefs and self‐efficacy about healthy *sleep behaviours*, *daytime sleepiness* at school and internalising symptoms. These findings bear implications for researchers and practitioners in the US and globally about universal prevention services for the commonly “sleep‐deprived” adolescents our schools serve.

### 4.1 The Positive Effect of *SLEEPS* on Middle School Students' Sleep

Consistent with previous studies (Díaz‐Morales et al. 2012), improvements were seen in adolescents' *sleep‐related beliefs*, lending partial support to the efficacy of the strategic education components of *SLEEPS* and TPB that sought to increase adolescents' meta‐cognitive perceptions and beliefs about the consequences of insufficient sleep and the benefits of healthy *sleep behaviours* (Cook et al. 2015; Zhang et al. 2025). Based on TPB (Worthington 2021), one's attitude toward sleep is determined by their belief that healthy sleep would lead to positive outcomes (e.g., less *daytime sleepiness*) with subjective values (e.g., happiness). Hence, fostering adolescents' positive beliefs is a prerequisite to supportive attitudes about sleep. Also, a positive effect was observed for adolescents' self‐efficacy about healthy *sleep behaviours*, which corroborated findings in prior studies. Several trials and reviews have highlighted that motivational interview (MI) can be used as a standalone or additive strategy to strengthen the effectiveness of sleep programs for adolescents self‐doubting their ability to initiate/maintain *sleep behaviour* change (e.g., Vrabec et al. 2025; Blake et al. 2019). These findings could inform researchers and schools about the utility of theory‐informed behaviour change strategies for developing or selecting sleep programs (Zhang, Cook, and Smith 2023).

Moreover, students receiving *SLEEPS* reported improvements in *daytime sleepiness*, a critical indicator of adolescent *sleep quality* known to impact their school functioning, physical and mental well‐being (Gaskin et al. 2024). This is further evidenced by the finding of the remedial effects of *SLEEPS* on adolescents' internalising symptoms. Given the prevalence and profound impacts of internalising symptoms among adolescents during and after the pandemic, schools should consider integrating universal sleep programmes as their routine school‐based support to promote healthy sleep as a protective factor for all students (Corbin et al. 2024).

Nonetheless, in this study, *SLEEPS* did not show significant impacts on attitudes or subjective norms. Due to the COVID‐19 pandemic and school confinement, the authors had to change the delivery format of *SLEEPS* from in‐person to online. The literature during COVID‐19 suggested that online learning could lead to similar rates of knowledge acquisition compared to in‐person learning pre‐COVID (Yu and Yu 2023). However, the lack of socialisation and interaction with peers and adults would significantly undermine the effectiveness of the behaviour change strategies of *SLEEPS* that rely on social influence and interpersonal interaction. This could explain why the significant effect of *SLEEPS* was only found on adolescents' beliefs and self‐efficacy about healthy sleep, but not on their subjective norms or attitudes (Worthington 2021).

Moreover, no significant improvement was observed in adolescents' *sleep routine and environment*, technology use, academic engagement and motivation. This is consistent with prior research showing sleep environment, routines and electronic use are the most resistant to change (Hedin et al. 2020). COVID‐19 has exacerbated the environmental distress for adolescents, parents, and educators in school and home, which could have undermined the effectiveness of *SLEEPS* on these outcomes (Audisio et al. 2022). Considering the prevalence of sleep problems has increased in the post‐pandemic era (Hassan et al. 2024), the authors call for researchers to use system science to refine and amplify *SLEEPS* by strengthening behaviour change strategies targeting factors across various socio‐ecological levels to improve adolescents' *sleep behaviours* and environments at school, home, community and beyond (Azad et al. 2024; Heemskerk et al. 2025; Meltzer et al. 2021).

### 4.2 Baseline Outcome Moderating the Effect of *SLEEPS*

Research suggests that students with diverse backgrounds or baseline needs respond differently to the same program (Zhang, Cook, Azad, et al. 2023). Similarly, the treatment effect heterogeneity of *SLEEPS* was observed wherein the students holding weaker beliefs about sleep at baseline improved more after receiving *SLEEPS* than peers holding stronger beliefs at baseline. This finding aligns with prior studies that highlight disparities in the sleep health of adolescents from marginalised or disadvantaged backgrounds (Billings et al. 2021). For instance, review studies have identified that disparity in sleep‐related cognitive factors (e.g., beliefs, attitudes; Grandner et al. 2016) can explain racial‐ethnic variability in the sleep health of adolescents from marginalised backgrounds. Relatedly, another crucial factor is the disparity in disadvantaged adolescents' actual sleep hygiene (e.g., lack of healthy sleep habits or environmental factors; Guglielmo et al. 2018). From a system‐level perspective, these factors and disparities were attributable to numerous systemic and structural inequities, such as poverty, homelessness, noisy and overcrowded environments (Falbe et al. 2015). School professionals should deliver school‐based sleep programs to promote equity in sleep hygiene among their students, especially those who are struggling with sleep problems. This is especially crucial given the increasingly diverse student bodies in schools and the long‐standing disparities in sleep hygiene among adolescents internationally (Billings et al. 2021).

### 4.3 Limitations and Future Directions

The authors acknowledge some limitations in this study that warrant cautious interpretation of certain findings. First, school context is associated with adolescents' behavioural health and the implementation of universal intervention programs like *SLEEPS* (Pauling et al. 2023). Based on this study, future studies could invest in a more comprehensive and diverse sample with students (at level 1) nested in different school contexts (at level 2) from different districts/regions/countries (at level 3) with varied school start times or sleep‐related policies. This sample could power multisite CRTs with three‐level ML‐ANCOVA to explore if multiple dimensions of diversity (e.g., race, poverty level, cultural norms) moderate the effectiveness of *SLEEPS* (Grandner et al. 2016). The larger sample could also enable more sensitive detection of the interaction between *SLEEPS* and adolescents' demographics that were found non‐significant in this study.

Third, to ensure the practicality of this study, the outcomes measured in this study were determined based on the theory of change of *SLEEPS* and school stakeholder inputs. However, we did not measure directly adolescents' self‐reported *sleep quality* at night. We call for future studies to extend from ours by using direct and multi‐informant measures of *sleep quality* (e.g., the self‐report and parent‐report forms of Pittsburgh Sleep Quality Index; Fabbri et al. 2021). This direct assessment of *sleep quality* by multiple informants (e.g., adolescents and parents) can improve the internal validity of the program evaluation (De Los Reyes et al. 2015). More importantly, their findings may further our understanding of which dimensions of *sleep quality* are improved by *SLEEPS*, resulting in less *daytime sleepiness* (Fabbri et al. 2021).

Moreover, the pandemic social isolation imposed unexpected restrictions on this study. The authors call for post‐pandemic replication studies with in‐person delivery of *SLEEPS* to explore unconfounded estimates on adolescents' sleep outcomes contingent on socialisation. In those studies, the process of development, revision, implementation and evaluation planning of *SLEEPS* should proactively and comprehensively involve school stakeholders via co‐creation and participatory action research (Heemskerk et al. 2025). This could ensure the development and adaptation align with the inputs and needs of the participants and school stakeholders in post‐pandemic (Vandendriessche et al. 2023, 2025; Inhulsen et al. 2022). In conjunction with using co‐creative and participatory action research, future studies about *SLEEPS* should take a broader and positive conceptualization of sleep health and system science approaches (Heemskerk et al. 2025). For instance, researchers should amplify the scope of *SLEEPS* by incorporating more aspects of the socio‐ecological system for adolescents (e.g., school‐home‐community contexts; Meltzer et al. 2021) and by measuring strength‐oriented positive dimensions of sleep health (e.g., sleep‐related resilience; Buysse 2014). To achieve these aims, system science methods (e.g., action scales models, causal loop diagrams; Heemskerk et al. 2025) should be embedded to create a system‐wide holistic action plan for both changes in adolescents' sleep hygiene and systemic factors across various socio‐ecological levels.

## 5 Conclusion

This study lends preliminary evidence supporting the effectiveness of *SLEEPS* as a universal intervention program for middle school students' sleep and related outcomes. It showed the importance of theory‐informed change mechanisms for guiding the effective development of universal sleep programs. Moreover, there is emerging evidence supporting the advantage of integrated universal programs and the utility of system science approaches for long‐lasting improvement in adolescents' sleep. Therefore, the authors call for future research to integrate *SLEEPS* into other universal programs with system science approaches to promote the behavioural, mental health and academic outcomes of the “whole child” in our increasingly diverse student population.

## Author Contributions

**Yanchen Zhang:** investigation, writing – original draft, methodology, validation, visualization, writing – review and editing, software, formal analysis, data curation, conceptualization. **Kayla T. Johnson:** writing – review and editing, resources. **Kyla Wahlstrom:** funding acquisition, investigation, project administration, supervision, resources, writing – review and editing, conceptualization. **Rachel Widome:** supervision, resources, writing – review and editing, project administration, funding acquisition, investigation, conceptualization. **Corinne Hamlin:** investigation, data curation, resources, project administration, software, writing – review and editing. **Andrew J. Barnes:** investigation, funding acquisition, supervision, resources, project administration, writing – review and editing, conceptualization.

## Ethics Statement

This study was reviewed and approved by the Institutional Review Board (IRB) of the principal investigator's university (STUDY00004280). All procedures performed in studies involving human participants were in accordance with the 1964 Helsinki declaration and its later amendments or comparable ethical standards.

## Consent

Informed consent was obtained from all participants in the study.

## Conflicts of Interest

The authors declare no conflicts of interest.

## Supporting information


Data S1.

Data S2.

Data S3.

Data S4.

Data S5.

## Data Availability

The data that support the findings of this study are openly available in Open Science Framework at https://osf.io/9skf5/, reference number https://doi.org/10.17605/OSF.IO/38MXT.

## References

[jsr70123-bib-0001] Audisio, K. , H. Lia , N. B. Robinson , et al. 2022. “Impact of the COVID‐19 Pandemic on Non‐COVID‐19 Clinical Trials.” Journal of Cardiovascular Development and Disease 9, no. 1: 19. 35050229 10.3390/jcdd9010019 PMC8781416

[jsr70123-bib-0002] Azad, G. F. , I. Taormina , V. Herrera , and Y. Zhang . 2024. “Communication Training Within Partners in School: Feasibility, Acceptability, and Usability.” Journal of Educational and Psychological Consultation 34, no. 3: 239–264. 39148644 10.1080/10474412.2024.2341382 PMC11323131

[jsr70123-bib-0003] Baraldi, A. N. , and C. K. Enders . 2010. “An Introduction to Modern Missing Data Analyses.” Journal of School Psychology 48, no. 1: 5–37. 20006986 10.1016/j.jsp.2009.10.001

[jsr70123-bib-0004] Billings, M. E. , R. T. Cohen , C. M. Baldwin , et al. 2021. “Disparities in Sleep Health and Potential Intervention Models: A Focused Review.” Chest 159, no. 3: 1232–1240. 33007324 10.1016/j.chest.2020.09.249 PMC7525655

[jsr70123-bib-0005] Blake, M. J. , M. D. Latham , L. M. Blake , and N. B. Allen . 2019. “Adolescent‐Sleep‐Intervention Research: Current State and Future Directions.” Current Directions in Psychological Science 28, no. 5: 475–482.

[jsr70123-bib-0006] Brysbaert, M. , and D. Debeer . 2025. “How to Run Linear Mixed Effects Analysis for Pairwise Comparisons? A Tutorial and a Proposal for the Calculation of Standardized Effect Sizes.” Journal of Cognition 8, no. 1: 5. 39803174 10.5334/joc.409 PMC11720698

[jsr70123-bib-0007] Busch, V. , T. M. Altenburg , I. A. Harmsen , and M. J. Chinapaw . 2017. “Interventions That Stimulate Healthy Sleep in School‐Aged Children: A Systematic Literature Review.” European Journal of Public Health 27, no. 1: 53–65. 28177474 10.1093/eurpub/ckw140

[jsr70123-bib-0056] Buysse, D. J. 2014. “Sleep Health: Can we Define it? Does it Matter?” Sleep 37, no. 1: 9–17. 24470692 10.5665/sleep.3298 PMC3902880

[jsr70123-bib-0008] CDC, Division of Population Health, National Center for Chronic Disease Prevention and Health Promotion . 2020. Sleep in Middle and High School Students. Centers for Disease Control and Prevention. https://www.cdc.gov/healthyschools/features/students‐sleep.htm.

[jsr70123-bib-0009] Cook, C. R. , A. R. Lyon , D. Kubergovic , D. Browning Wright , and Y. Zhang . 2015. “A Supportive Beliefs Intervention to Facilitate the Implementation of Evidence‐Based Practices Within a Multi‐Tiered System of Supports.” School Mental Health 7: 49–60.

[jsr70123-bib-0010] Corbin, C. M. , Y. Zhang , M. G. Ehrhart , J. Locke , and A. R. Lyon . 2024. “Testing an Organizational Implementation Process Model Related to Teachers' Implementation‐Related Attitudes and Behaviors: A Multilevel Mediation Analysis.” Prevention Science 25, no. 7: 1053–1064. 39271598 10.1007/s11121-024-01722-6

[jsr70123-bib-0011] Dang, Z. , C. Deng , X. Yang , K. Wei , and H. Huang . 2021. “Nearest Neighbor Matching for Deep Clustering.” In Proceedings of the IEEE/CVF Conference on Computer Vision and Pattern Recognition, 13693–13702. IEEE Computer Society Press.

[jsr70123-bib-0012] De Los Reyes, A. , T. M. Augenstein , M. Wang , et al. 2015. “The Validity of the Multi‐Informant Approach to Assessing Child and Adolescent Mental Health.” Psychological Bulletin 141, no. 4: 858. 25915035 10.1037/a0038498 PMC4486608

[jsr70123-bib-0013] Dewald, J. F. , A. M. Meijer , F. J. Oort , G. A. Kerkhof , and S. M. Bögels . 2010. “The Influence of Sleep Quality, Sleep Duration and Sleepiness on School Performance in Children and Adolescents: A Meta‐Analytic Review.” Sleep Medicine Reviews 14, no. 3: 179–189. 20093054 10.1016/j.smrv.2009.10.004

[jsr70123-bib-0014] Díaz‐Morales, J. F. , P. D. Prieto , C. E. Barreno , M. J. C. Mateo , and C. Randler . 2012. “Sleep Beliefs and Chronotype Among Adolescents: The Effect of a Sleep Education Program.” Biological Rhythm Research 43, no. 4: 397–412.

[jsr70123-bib-0015] DiPerna, J. C. 2005. “Academic Enablers and Student Achievement: Implications for Assessment and Intervention Services in the Schools.” Psychology in the Schools 43: 7–17.

[jsr70123-bib-0016] Drake, C. , C. Nickel , E. Burduvali , T. Roth , C. Jefferson , and B. Pietro . 2003. “The Pediatric Daytime Sleepiness Scale (PDSS): Sleep Habits and School Outcomes in Middle‐School Children.” Sleep 26, no. 4: 455–458. 12841372

[jsr70123-bib-0017] Dunn, T. J. , T. Baguley , and V. Brunsden . 2014. “From Alpha to Omega: A Practical Solution to the Pervasive Problem of Internal Consistency Estimation.” British Journal of Psychology 105, no. 3: 399–412. 24844115 10.1111/bjop.12046

[jsr70123-bib-0018] Duong, M. T. , E. J. Bruns , K. Lee , et al. 2021. “Rates of Mental Health Service Utilization by Children and Adolescents in Schools and Other Common Service Settings: A Systematic Review and Meta‐Analysis.” Administration and Policy in Mental Health and Mental Health Services Research 48: 420–439. 32940884 10.1007/s10488-020-01080-9

[jsr70123-bib-0019] Eldredge, L. K. B. , C. M. Markham , R. A. Ruiter , M. E. Fernández , G. Kok , and G. S. Parcel . 2016. Planning Health Promotion Programs: An Intervention Mapping Approach. John Wiley & Sons.

[jsr70123-bib-0020] Fabbri, M. , A. Beracci , M. Martoni , D. Meneo , L. Tonetti , and V. Natale . 2021. “Measuring Subjective Sleep Quality: A Review.” International Journal of Environmental Research and Public Health 18, no. 3: 1082. 33530453 10.3390/ijerph18031082 PMC7908437

[jsr70123-bib-0054] Falbe, J. , K. K. Davison , R. L. Franckle , et al. 2015. “Sleep Duration, Restfulness, and Screens in the Sleep Environment.” Pediatrics 135, no. 2: e367–e375. 25560435 10.1542/peds.2014-2306 PMC4306800

[jsr70123-bib-0021] Francis, J. J. , M. P. Eccles , M. Johnston , et al. 2004. Constructing Questionnaires Based on the Theory of Planned Behavior. A Manual for Health Services Researchers. Centre for Health Services Research, University of Newcastle upon Tyne. www.rebeqi.org.

[jsr70123-bib-0022] Gaskin, C. J. , C. Venegas Hargous , L. D. Stephens , et al. 2024. “Sleep Behavioral Outcomes of School‐Based Interventions for Promoting Sleep Health in Children and Adolescents Aged 5 to 18 Years: A Systematic Review.” SLEEP Advances 5, no. 1: zpae019. 38584765 10.1093/sleepadvances/zpae019 PMC10996385

[jsr70123-bib-0023] Grandner, M. A. , N. J. Williams , K. L. Knutson , D. Roberts , and G. Jean‐Louis . 2016. “Sleep Disparity, Race/Ethnicity, and Socioeconomic Position.” Sleep Medicine 18: 7–18. 26431755 10.1016/j.sleep.2015.01.020 PMC4631795

[jsr70123-bib-0024] Guglielmo, D. , J. A. Gazmararian , J. Chung , A. E. Rogers , and L. Hale . 2018. “Racial/Ethnic Sleep Disparities in US School‐Aged Children and Adolescents: A Review of the Literature.” Sleep Health 4, no. 1: 68–80. 29332684 10.1016/j.sleh.2017.09.005 PMC5771439

[jsr70123-bib-0025] Hale, L. , and J. M. Dzierzewski . 2024. “Screens and Sleep Health—It's Almost Bedtime, Time to Put Your Phone Away.” JAMA Pediatrics 178, no. 10: 963–964. 39102250 10.1001/jamapediatrics.2024.2757

[jsr70123-bib-0026] Hassan, N. F. , S. A. Ail , A. S. Jabbar , and H. A. Abdulameer . 2024. “Post‐Pandemic Sleep Disorders: Understanding Insomnia in the Aftermath of COVID‐19.” Central Asian Journal of Medical and Natural Science 5, no. 2: 72–81.

[jsr70123-bib-0027] Hayes, A. F. , and N. J. Rockwood . 2020. “Conditional Process Analysis: Concepts, Computation, and Advances in the Modeling of the Contingencies of Mechanisms.” American Behavioral Scientist 64, no. 1: 19–54.

[jsr70123-bib-0028] Hedin, G. , A. Norell‐Clarke , P. Hagell , H. Tønnesen , A. Westergren , and P. Garmy . 2020. “Facilitators and Barriers for a Good Night's Sleep Among Adolescents.” Frontiers in Neuroscience 14: 92. 32116531 10.3389/fnins.2020.00092 PMC7019014

[jsr70123-bib-0029] Heemskerk, D. M. , M. M. van Stralen , J. T. Piotrowski , C. M. Renders , and V. Busch . 2025. “Developing a Whole Systems Action Plan Promoting Dutch Adolescents' Sleep Health.” International Journal of Behavioral Nutrition and Physical Activity 22, no. 1: 33. 40097968 10.1186/s12966-025-01711-0 PMC11917006

[jsr70123-bib-0030] Inhulsen, M. B. M. , V. Busch , and M. M. van Stralen . 2022. “Effect Evaluation of a School‐Based Intervention Promoting Sleep in Adolescents: A Cluster‐Randomized Controlled Trial.” Journal of School Health 92, no. 6: 550–560. 35315076 10.1111/josh.13175 PMC9314837

[jsr70123-bib-0031] Kohyama, J. 2024. “Re‐Evaluating Recommended Optimal Sleep Duration: A Perspective on Sleep Literacy.” Children 11, no. 9: 1098. 39334630 10.3390/children11091098 PMC11429570

[jsr70123-bib-0055] Meltzer, L. J. , A. A. Williamson , and J. A. Mindell . 2021. “Pediatric Sleep Health: It Matters, and so does how we Define it.” Sleep Medicine Reviews 57: 101425. 33601324 10.1016/j.smrv.2021.101425 PMC9067252

[jsr70123-bib-0032] Mitchell, T. B. , J. L. Cooley , C. Cummings , et al. 2024. “Latent Profiles of Sleep Disturbance and Impairment in Elementary School‐Age Youth: Concurrent and Longitudinal Associations With Emotional, Behavioral, and Academic Functioning.” Journal of Pediatric Psychology 49, no. 3: 153–163. 38013220 10.1093/jpepsy/jsad077

[jsr70123-bib-0033] Osborne, J. W. , and E. Waters . 2002. “Four Assumptions of Multiple Regression That Researchers Should Always Test.” Practical Assessment, Research & Evaluation 8, no. 1: 2.

[jsr70123-bib-0034] Owens, J. A. , K. Belon , and P. Moss . 2010. “Impact of Delaying School Start Time on Adolescent Sleep, Mood, and Behavior.” Archives of Pediatrics & Adolescent Medicine 164, no. 7: 608–614. 20603459 10.1001/archpediatrics.2010.96

[jsr70123-bib-0035] Pauling, S. , C. Cook , K. Pekel , M. Larson , and Y. Zhang . 2023. “A Cross‐Sectional Survey of School Administrators' Implementation of Evidence‐Based Practices and Programs: Training, Knowledge, and Perceived Barriers.” Leadership and Policy in Schools 22, no. 3: 676–694.

[jsr70123-bib-0036] Pegado, A. , M. J. Alvarez , and M. S. Roberto . 2023. “The Role of Behaviour‐Change Theory in Sleep Interventions With Emerging Adults (Aged 18–29 Years): A Systematic Review and Meta‐Analysis.” Journal of Sleep Research 32, no. 5: e13877. 36922157 10.1111/jsr.13877

[jsr70123-bib-0037] Rigney, G. , A. Watson , J. Gazmararian , and S. Blunden . 2021. “Update on School‐Based Sleep Education Programs: How Far Have We Come and What Has Australia Contributed to the Field?” Sleep Medicine 80: 134–157. 33607553 10.1016/j.sleep.2021.01.061

[jsr70123-bib-0038] Sanetti, L. M. H. , and T. R. Kratochwill . 2011. “An Evaluation of the Treatment Integrity Planning Protocol and Two Schedules of Treatment Integrity Self‐Report: Impact on Implementation and Report Accuracy.” Journal of Educational and Psychological Consultation 21, no. 4: 284–308.

[jsr70123-bib-0039] Storfer‐Isser, A. , M. K. Lebourgeois , J. Harsh , C. J. Tompsett , and S. Redline . 2013. “Psychometric Properties of the A Dolescent S Leep H Ygiene S Cale.” Journal of Sleep Research 22, no. 6: 707–716. 23682620 10.1111/jsr.12059 PMC3752307

[jsr70123-bib-0040] Tonetti, L. , A. Andreose , V. Bacaro , M. Grimaldi , V. Natale , and E. Crocetti . 2022. “Different Effects of Social Jetlag and Weekend Catch‐Up Sleep on Well‐Being of Adolescents According to the Actual Sleep Duration.” International Journal of Environmental Research and Public Health 20, no. 1: 574. 36612896 10.3390/ijerph20010574 PMC9819690

[jsr70123-bib-0041] Vandendriessche, A. , B. Deforche , K. Dhondt , T. M. Altenburg , and M. Verloigne . 2023. “Combining Participatory Action Research With Intervention Mapping to Develop and Plan the Implementation and Evaluation of a Healthy Sleep Intervention for Adolescents.” Health Promotion Perspective 13, no. 4: 316–329. 10.34172/hpp.2023.37 PMC10790120 38235009

[jsr70123-bib-0042] Vandendriessche, A. , B. Deforche , K. Dhondt , M. Verloigne , and J. Van Cauwenberg . 2025. “Effect Evaluation of a Participatory Developed School‐Based Healthy Sleep Intervention for Adolescents.” Health Psychology 44: 380–390. 39869691 10.1037/hea0001443

[jsr70123-bib-0043] Vrabec, A. , M. A. Milligan , K. M. Antshel , and K. M. Kidwell . 2025. “A Meta‐Analytic Review of Cognitive Behavior Therapy and Motivational Interviewing for Adolescent and Young Adult Sleep Concerns.” Clinical Child Psychology and Psychiatry 30, no. 2: 386–401. 39666334 10.1177/13591045241308983

[jsr70123-bib-0044] Wahlstrom, K. , B. Dretzke , M. Gordon , K. Peterson , K. Edwards , and J. Gdula . 2014. “Examining the Impact of Later School Start Times on the Health and Academic Performance of High School Students: A Multi‐Site Study.” Center for Applied Research and Educational Improvement. University of Minnesota. St Paul, MN.

[jsr70123-bib-0045] Wolfson, A. R. , M. A. Carskadon , C. Acebo , et al. 2003. “Evidence for the Validity of a Sleep Habits Survey for Adolescents.” Sleep 26, no. 2: 213–216. 12683482 10.1093/sleep/26.2.213

[jsr70123-bib-0046] Worthington, A. K. 2021. “Theory of Planned Behavior.” In Persuasion Theory in Action: An Open Educational Resource. Pressbooks.

[jsr70123-bib-0047] Yu, Z. , and L. Yu . 2023. “Examining Factors That Influence Learner Retention in MOOCs During the COVID‐19 Pandemic Time.” SAGE Open 13, no. 2: 21582440231175371. 37275328 10.1177/21582440231175371 PMC10230298

[jsr70123-bib-0048] Zhang, Y. , C. R. Cook , G. F. Azad , et al. 2023. “A Pre‐Implementation Enhancement Strategy to Increase the Yield of Training and Consultation for School‐Based Behavioral Preventive Practices: A Triple‐Blind Randomized Controlled Trial.” Prevention Science 24, no. 3: 552–566. 36367633 10.1007/s11121-022-01464-3 PMC10258873

[jsr70123-bib-0049] Zhang, Y. , C. R. Cook , and B. Smith . 2023. “PurposeFull People SEL and Character Education Program: A Cluster Randomized Trial in Schools Implementing Tier 1 PBIS With Fidelity.” School Mental Health 15: 985–1002.

[jsr70123-bib-0050] Zhang, Y. , L. Fallon , M. Larson , D. Browning Wright , C. R. Cook , and A. R. Lyon . 2025. “Associations Among Educators' Beliefs, Intervention Fidelity, and Student Outcomes in School‐Wide Positive Behavior Interventions, and Supports: A School‐Level Moderated Mediation Analysis.” School Psychology 40, no. 4: 431–444. 38695806 10.1037/spq0000615

