# Supplementary material for: A Cluster Randomised Trial of a School‐Based Universal Intervention Program for Middle School Students' Sleep and Related Outcomes
Source: J Sleep Res. 2025 Jun 18;35(1):e70123. doi: 10.1111/jsr.70123 (PMC12856133; doi:10.1111/jsr.70123)
Supplement: Supplementary file 1 — Data S1. [file JSR-35-e70123-s003.docx]

# Supplementary material 1: Details of SLEEPS

## Free Access to SLEEPS

The final version of this iteratively designed intervention is available at <http://z.umn.edu/sleepbetter> and is free for non-commercial use, with attribution to the authors, under Creative Commons 4.0 license.

## Iterative Development and Revision of the SLEEPS Program

The program development procedure followed the Intervention Mapping (IM) approach by going through a 6-step iterative path (problem identification, logic model of change, program design, program production, implementation plan, and evaluation; Bartholomew et al., 2016). For SLEEPS program development and refinement, we first conducted a series of co-productive and participatory qualitative studies - (a) focus groups with key school stakeholders and (b) an expert summit in order to engage in an information-gathering process that will inform re-visions of SLEEPS and refine it for subsequence feasibility testing, and (c) an intervention feasibility/acceptability demonstration to gather systematic feedback from key stakeholder groups.

First, we held focus groups with key school stakeholders to collect information about needs assessment of areas to focus on regarding middle school students' healthy sleep behaviors and related outcomes, actionable information to inform strategic efforts to develop the initial version of SLEEPS that is developmentally appropriate and feasible, contextual considerations for curriculum delivery in middle school settings, etc. Two focus group sessions were held separately with each of the groups: (1) teachers, (2) administrators (district and principals), (3) students, and (4) parents. Each group had 6 to 8 participants, as groups of this size are sufficient to provide a satisfactory range of perspectives, reach saturation, and make it difficult for certain individuals to dominate the discussion (Krue-ger & Casey, 2008; Morgan, 1996). We followed focus group procedures outlined by Morgan (1996, 1998) and Krueger and Casey (2008). Led by the project investigators, a semi-structured discussion format was used to gather qualitative information from stakeholders in response to a carefully predetermined set of probing questions to gather input about the SLEEPS, target outcomes, its intervention components, curriculum delivery model (where, when, how many lessons, length of lessons), and addressing practical issues that are likely to arise (e.g., students missing lessons, teacher absences).

Building off of focus group findings, we then hosted an Delphi Expert Summit (Jorm, 2015) to gather in-depth recommendations from national and regional sleep experts to evaluate our theory of change, receive input about the specific content and activities associated with each of the intervention components to ensure integrity with scientific research, and determine methods for enhancing the usability of SLEEPS curriculum to facilitate its successful integration into middle school settings.

Finally, building off the findings from the two prior studies, we continued to iteratively revise SLEEPS by conducting an intervention feasibility/acceptability demonstration to gather systematic feedback from key stakeholder groups (e.g., students, caregivers, teachers, school leaders, experts) using mixed methods. Specifically, relevant components of SLEEPS were introduced to students, caregivers and teachers to gather feasibility and acceptability ratings, with quantitative data tallied in real time and followed by focus groups to collect qualitative explanations for high/low ratings and solicit recommendations to revise specific components of SLEEPS. This two-part process (informational material followed by actual demonstration) will allow for the evaluation of participants' perceptions of the SLEEPS curriculum based on its actual delivery (Suh & Lee, 2005). Additionally, we followed up with students and caregivers two weeks after the demonstrations to examine the impact of SLEEPS on their attitudes, knowledge, and behavior about sleeps. Acceptability, Likely Effectiveness, Feasibility, and Appropriateness Questionnaire (ALFA-Q) was used to assess the social validity of specific segments of the SLEEPS, as well as for the intervention as a whole.

## Training for Educators To Implement SLEEPS

The training employed a "Tell-Show-Do-Feedback" model wherein teachers were provided with rationales and an opportunity to see the curriculum delivered by the authors, after which the teachers practiced implementing the curriculum. Then, the authors supported teachers in creating an implementation plan about when and how to implement *SLEEPS* and identify implementation barriers with solutions. Also, the treatment group was asked to complete a lesson implementation plan before each lesson.

# Theory-Informed Behavior Change Strategies of SLEEPS

## Strategic Education to Improve Beliefs and Attitudes

Delivery of strategic education has been shown to result in effective behavior when there is an active learning process in which individuals acquire knowledge and beliefs through interaction and reflection (El Tannir, 2002). Attitudes towards the behavior have been shown to arise from a combination of beliefs about the consequences of behavior (behavioral beliefs) and evaluations of the associated consequences (outcome evaluations). Many middle school students have insufficient knowledge and beliefs about the importance of sleep and the consequences of sleep problems on their wellbeing and performance (McDowell et al., 2016; Noland et al., 2009). Thus, to influence students’ attitudes about healthy sleep behaviors, SLEEPS incorporates strategic education that focuses on increasing students’ knowledge and beliefs of the link between sleep, brain functioning, and their own physical, emotional, and academic wellbeing. Students will also learn about behaviors (e.g., nighttime screen time, caffeine consumption) and consequences (e.g., daytime sleepiness, moodiness, attractiveness) associated with insufficient sleep, as well as the positive consequences of getting sufficient sleep. Last, students will receive strategic education about healthy sleep behaviors (e.g., mindfulness and relaxation methods for stress reduction and insomnia; having a bedtime routine; maintaining consistent sleep-wake times on weekends) that promote good sleep quality, adequate sleep duration, and daytime alertness (LeBourgeois et al., 2005). As a summative activity, students will participate in a values affirmation activity (see Cohen & Sherman, 2014; McQueen & Klein, 2006), which is an evidence-based strategy shown to promote behavior change. Values affirmation activities have been shown to decrease defensive attitudes toward change by boosting self-images or self-worth (Walton & Cohen, 2011). Specifically, the values activity will involve students identifying core values as a student and linking them to healthy sleep behaviors. This activity will focus on enhancing students’ intrinsic positive attitudes towards healthy sleep. By incorporating student feedback in its development, SLEEPS was designed to be developmentally appropriate and relevant, maximizing student engagement and participation in lessons.

As discussed above, caregivers also have insufficient knowledge and beliefs when it comes to sleep and often do not know how to support their child’s healthy sleep behaviors (Owens & Jones, 2011; Schreck & Richdale, 2011). The universal level caregiver support involves low-intensity strategies to influence caregivers support within the home setting, which consists of electronically delivered educational resources (tip sheets), brief videos of students talking to parents about the importance of sleep, and weekly text prompts that include “pearls of wisdom” to remind caregivers to take an active role in supporting their child’s healthy sleep habits. The caregiver resources will indirectly impact youth’s attitudes by influencing how caregivers talk about the importance of sleep with their child, as well as will impact one of the other TPB components: youth’s subjective norms about healthy sleep.

## Motivational Interviewing to Enhance Perceived Behavioral Control

SLEEPS adapts Motivational Interviewing (MI; Rollnick & Miller, 1995), an evidence-based intervention to improve health-related behavior in adolescents (Miller & Rose, 2009). MI adopts an empathic communication style and uses person-centered communication strategies to promote self-efficacy through eliciting and supporting hope, optimism, and the feasibility of making changes in one’s behavior (Miller & Rollnick, 2012; Rollnick et al., 2016; Lundahl et al., 2010). MI has been adapted and tested for use with a variety of child behaviors in clinics and classrooms (e.g., Family Check-Up, Classroom Check-Up), yielding strong evidence for effects on parents and teachers (Connell, Dishion, Yasui, & Kavanagh, 2007; Reinke et al., 2012). Most recently, MI has been shown in meta-analyses and systematic reviews to alter adolescent behavior with regard to substance use (Tanner-Smith & Lipsey, 2015) and health-related behaviors (Asarnow et al., 2015; Barnes & Gold, 2012). MI also facilitates maintenance of behavior change and prevent relapse to old habits (Babor et al., 2007). Ample evidence documents the impact of MI on adolescent health behaviors (Cushing et al., 2014; Gayes & Steele, 2014; Jensen et al., 2011) and use of MI in the context of interactive groups (D’Amico et al., 2015). Given MI’s strong empirical support, SLEEPS adapts MI techniques to increase perceived behavioral control among middle school students (see Figure 2). An empathic, supportive, and person-centered style were integrated into the SLEEPS curriculum and embedded activities to elicit self-motivational statements and promote students’ self-efficacy to engage in healthy sleep habits. For example, to enhance perceived behavioral control, students anticipate barriers that may arise when attempting to engage in healthy sleep behaviors; brainstorm ways to address those barriers; answer standard MI “ruler” questions (e.g., “On a scale of 1-10, how confident are you in your ability to engage? Why that score and not a lower one? What would move your score to a higher number?”); and engage in group discussion to evoke “change talk” about what is needed to increase their ratings. Additionally, to impact perceived behavioral control by eliciting change talk, students recall and share with others positive changes they have made in their lives, highlighting their capability to alter their behavior. Moreover, group-based discussions focus on helping students shift their perceptions of their own behavior from external (e.g., “Nothing I can do because have too much homework”) to an internal (e.g., “I can choose to turn off my phone and put it in the other room”) locus of control. Consistent with prior research, the main goal is for students to recognize that they have control over their sleep by altering their own behavior. SLEEPS emphasizes MI communicative style (e.g., questions and reflective statements that elicit change talk), and MI strategies (ruler questions, pro/con lists) were embedded throughout the curriculum, to promote students’ self-efficacy. Last, MI strategies were integrated into the booster sessions which focuses on supporting maintenance of behavior change.

## Social Psychology Influence Techniques to Increase Subjective Norms

Neither the Strategic Education or MI components address the third determinant of behavioral intentions: subjective norms—a gap which is especially problematic because schools are inherently social contexts where behavior is influenced by actions and opinions of others (Gehlbach, 2010). The discipline of social psychology has demonstrated that people’s decisions and behaviors are affected by others, and that basic social psychological techniques can be used to influence healthy choices (Pratkanis, 2007). With SLEEPS, we aim to shift middle school students’ subjective norms regarding healthy sleep behaviors by adapting two evidence-informed social psychological influence techniques: (1) social proofing and (2) inducing cognitive dissonance. Consistent with the TPB, the SLEEPS curriculum aims to change middle school students’ subjective norms so they understand that other admired and/or respected others in their lives (peers and adults) also value and strive to get good sleep.

Social proofs are grounded in decades of scientific research demonstrating that individuals make decisions partially based on the behavior of others judged to be respected, credible, and/or similar to themselves (Cialdini & Goldstein, 2004). Carefully-crafted social proofing messages about social norms (e.g., data or testimonials describing the behavior or attitudes of others) can be effective in reducing a variety of problem behaviors, including alcohol use (Perkins et al., 1999), illegal drug use (Perkins, 1994), cigarette smoking (Chassin et al., 1984), and eating disordered behaviors (Bergstrom & Neighbors, 2006). Providing normative information – a social proofing strategy – is most effective when people are given information about the current behavior of individuals with whom they closely identify (Cialdini, Reno, & Kalgren, 1990). Another social proofing strategy, testimonial evidence, has also been found to be effective, especially when testimonials speak to the usefulness of the specific behavior in solving relevant problems (Abrams et al., 1990; Berkowitz, 2003). Social proofs, such as normative and testimonial information, are interspersed throughout SLEEPS (see Appendix D.5 for example testimonials). Moreover, the caregiver educational resources were crafted to include testimonials from other parents and students to reinforce social norms and expectations for sleep in the home environment.

A second category of social psychology influence techniques involves strategies to induce cognitive dissonance, which operate on the premise that individuals strive for consistency between their attitudes and actions (Petrova, Cialdini, Sills, 2007). Thus, desired behaviors can be increased by evoking commitments that are active (rather than passive), public (rather than private), and voluntary (rather than coerced) (Cioffi & Garner, 1996; Pratkanis, 2007). Once individuals have made a commitment, their beliefs tend to shift to maintain consistency with their actions (Hausmann, Levine, & Higgins, 2007). This technique has been applied to a wide range of behaviors, including voting, dieting, fund-raising, and recycling (Greenwald et al., 1987; Spangenberg & Greenwalk, 2001). Another cognitive dissonance strategy capitalizes on the tendency for individuals to endorse and follow through with messages that they have freely advocated to others (Higgins & Rholes, 1978; Hausmann, Levine, & Higgins, 2007). For instance, college students randomly assigned to an experimental condition requiring them to write letters to younger students about how to overcome social and academic adversity showed significant improvements in school-related behaviors and achievement (Walton & Cohen, 2007). To impact subjective norms and facilitate commitment, student activities include actively talking with others about their commitments to engage in healthy sleep, as well as advocating a message endorsing the importance of healthy sleep to incoming students transitioning into middle school.

The educational tips targeting caregivers utilize social-proofing techniques to impact caregiver behavior and to facilitate improved norms and expectations in the home environment. Indeed, caregivers can have a powerful influence on youth’s sleep behaviors and outcomes by modeling and establishing social norms for healthy sleep behaviors (Meijer et al., 2016). Moreover, in addition to the educational tip sheets, additional caregiver resources were iteratively developed, such as brief videos of students talking to parents about the importance of sleep and weekly text prompts that include “pearls of wisdom” to remind caregivers to take an active role in modeling and creating norms within the home to promote healthy sleep habits. Behavioral research has shown that strategic messaging using social psychological strategies can significantly influence the behavior of recipients, including parents’ caregiving behavior in the home (Bigelow et al., 2008; York & Loeb, 2014).

A third social influence technique involves teachers employing an inter-dependent group contingency (IGC) to increase peer norms that promote student engagement during lessons and completion of practice-oriented homework. IGC capitalizes on positive peer pressure and reinforcement by dividing students into groups and having students within each group work together to earn a desired reward (Popkin & Skinner, 2003). Rewards are delivered to the entire group if each member meets the criterion (Gresham & Gresham, 1982). A meta-analysis on IGCs with children and youth demonstrated a pooled effect size of 2.88 across 35 studies, with diverse outcomes including exercise, positive behavior, work completion, and academic engagement (Little, Akin-Little, & O’Neill, 2015). SLEEPS utilizes in-class groups which are provided with practice-oriented homework assignments after each lesson. Each group is able to access desired rewards contingent upon members of the group completing and returning the homework assignment and maintaining engagement in lessons. Homework assignments involve specific behaviors relevant to healthy sleep, including monitoring sleep, bedtime routines, and using strategies to combat barriers to sleep.

# Reference

Abrams, D., Wetherell, M., Cochrane, S., Hogg, M. A., & Turner, J. C. (1990). Knowing what to think by knowing who you are: Self‐categorization and the nature of norm formation, conformity and group polarization. *British Journal of Social Psychology*, *29*(2), 97-119.

Asarnow J. R., Rozenman M., Wiblin J., & Zeltzer L. (2015). Integrated medical-behavioral care compared with usual primary care for child and adolescent behavioral health: A meta-analysis*. JAMA Pediatrics, 169*, 929–937.

Babor, T. F., McRee, B. G., Kassebaum, P. A., Grimaldi, P. L., Ahmed, K., & Bray, J. (2007). Screening, Brief Intervention, and Referral to Treatment (SBIRT): Toward a public health approach to the management of substance abuse. *Substance abuse*, *28*(3), 7–30. https://doi.org/10.1300/J465v28n03_03

Barnes, A. J., & Gold, M. A. (2012). Promoting healthy behaviors in pediatrics: Motivational interviewing. *Pediatrics in Review*, *33*(9), e57–e68. https://doi.org/10.1542/pir.33-9-e57

Bergstrom, R. L., & Neighbors, C. (2006). Body image disturbance and the social norms approach: An integrative review of literature. *Journal of Social and Clinical Psychology, 25, 995–1020*.

Berkowitz, A. D. (2003). Applications of social norms theory to other health and social justice issues. The social norms approach to preventing school and college age substance abuse: *A handbook for educators, counselors, and clinicians,* 1.

Bigelow, K. M., Carta, J. J., & Burke Lefever, J. (2008). Txt u ltr: Using cellular phone technology to enhance a parenting intervention for families at risk for neglect. *Child Maltreatment*, *13*(4), 362–367. https://doi.org/10.1177/1077559508320060

Chassin, L., Presson, C. C., Sherman, S. J., Corty, E., & Olshavsky, R. W. (1984). Predicting the onset of cigarette smoking in adolescents: A longitudinal study. *Journal of Applied Social Psychology*, *14*(3), 224-243.

Cialdini, R. B., & Goldstein, N. J. (2004). Social influence: Compliance and conformity. *Annual Review of Psychology*, *55*, 591-621.

Cialdini, R. B., Reno, R. R., & Kalgren, C. A. (1990). A focus theory of normative conduct: Recycling the concept of norms to reduce littering in public places. *Journal of Personality and Social Psychology*, *58*(6), 1015.

Cioffi, D., & Garner, R. (1996). On doing the decision: Effects of active versus passive choice on commitment and self-perception. *Personality and Social Psychology Bulletin*, *22*(2), 133-147.

Cohen, G. L., & Sherman, D. K. (2014). The psychology of change: Self-affirmation and social psychological intervention. *Annual Review of Psychology*, *65*, 333-371.

Connell, A. M., Dishion, T. J., Yasui, M., & Kavanagh, K. (2007). An adaptive approach to family intervention: Linking engagement in family-centered intervention to reductions in adolescent problem behavior. *Journal of Consulting and Clinical Psychology*, *75*(4), 568.

Cushing, C. C., Jensen, C. D., Miller, M. B., & Leffingwell, T. R. (2014). Meta-analysis of motivational interviewing for adolescent health behavior: Efficacy beyond substance use. *Journal of Consulting and ClinicalPsychology, 82*(6), 1212–1218. https://doi.org/10.1037/a0036912

D'Amico, E. J., Houck, J. M., Hunter, S. B., Miles, J. N., Osilla, K. C., & Ewing, B. A. (2015). Group motivational interviewing for adolescents: Change talk and alcohol and marijuana outcomes. *Journal of Consulting and Clinical Psychology*, *83*(1), 68–80. https://doi.org/10.1037/a0038155

*Educational Psychology Review*, *22*(3), 349-362.

Eldredge, L. K. B., Markham, C. M., Ruiter, R. A., Fernández, M. E., Kok, G., & Parcel, G. S. (2016). *Planning health promotion programs: an intervention mapping approach*. John Wiley & Sons.

El-Tannir, A. A. (2002). The corporate university model for continuous learning, training and development. *Education + Training*, *44*(2), 76-81.

Gayes, L. A., & Steele, R. G. (2014). A meta-analysis of motivational interviewing interventions for pediatric health behavior change. *Journal of Consulting and Clinical Psychology*, *82*(3), 521–535. https://doi.org/10.1037/a0035917

Gehlbach, H. (2010). The social side of school: Why teachers need social psychology.

Greenwald, A. G., Carnot, C. G., Beach, R., & Young, B. (1987). Increasing voting behavior by asking people if they expect to vote. *Journal of Applied Psychology*, *72*(2), 315.

Gresham, F. M., & Gresham, G. N. (1982). Interdependent, dependent, and independent group contingencies for controlling disruptive behavior. *The Journal of Special Education, 16*(1), 101-110. https://doi.org/10.1177/002246698201600110

Hausmann, L. R., Levine, J. M., & Higgins, E. T. (2007). Communication and group perception: Extending the ‘saying is believing' effect. *Group Processes & Intergroup Relations*, *11*(4), 539-554.

Higgins, E. T., & Rholes, W. S. (1978). “Saying is believing”: Effects of message modification on memory and liking for the person described. *Journal of Experimental Social Psychology*, *14*(4), 363-378.

Jensen, C. D., Cushing, C. C., Aylward, B. S., Craig, J. T., Sorell, D. M., & Steele, R. G. (2011). Effectiveness of motivational interviewing interventions for adolescent substance use behavior change: A meta-analytic review. *Journal of Consulting and Clinical Psychology*, *79*(4), 433–440. https://doi.org/10.1037/a0023992

Jorm, A. F. (2015). Using the Delphi expert consensus method in mental health research. *Australian & New Zealand Journal of Psychiatry*, *49*(10), 887-897.

LeBourgeois, M. K., Giannotti, F., Cortesi, F., Wolfson, A. R., & Harsh, J. (2005). The relationship between reported sleep quality and sleep hygiene in Italian and American adolescents. *Pediatrics*, *115*(1 Suppl), 257–265. https://doi.org/10.1542/peds.2004-0815H

Little, S. G., Akin-Little, A., & O'Neill, K. (2015). Group contingency interventions with children—1980-2010: A meta-analysis. *Behavior Modification, 39*(2), 322–341. https://doi.org/10.1177/0145445514554393

Lundahl, B. W., Kunz, C., Brownell, C., Tollefson, D., & Burke, B. L. (2010). A meta-analysis of motivational interviewing: Twenty-five years of empirical studies. *Research on Social Work Practice*, *20*(2), 137–160.

McQueen, A., & Klein, W. M. P. (2006). Experimental manipulations of self-affirmation: A systematic review. *Self and Identity, 5*, 289-354.

Meijer, A. M., Reitz, E., & Dekoviċ, M. (2016). Parenting matters: A longitudinal study into parenting and adolescent sleep. *Journal of sleep research*.

Miller, W. R., & Rollnick, S. (2012). *Motivational interviewing: Helping people change*. Guilford press.

Miller, W. R., & Rose, G. S. (2009). Toward a theory of motivational interviewing. *American psychologist*, *64*(6), 527.

Noland, H., Price, J. H., Dake, J., & Telljohann, S. K. (2009). Adolescents’ sleep behaviors and perceptions of sleep. *Journal of School Health, 79*(5), 224-230.

Owens, J. A., & Jones, C. (2011). Parental knowledge of healthy sleep in young children: Results of a primary care clinic survey. *Journal of Developmental and Behavioral Pediatrics, 32*, 447– 453.

Perkins, H. W. (1994). The contextual effect of secular norms on religiosity as moderator of student alcohol and other drug use. *Research in the Social Scientific Study of Religion*, *6*, 187-208.

Perkins, H. W., Meilman, P. W., Leichliter, J. S., Cashin, J. R., & Presley, C. A. (1999). Misperceptions of the norms for the frequency of alcohol and other drug use on college campuses. *Journal of American College Health*, *47*(6), 253-258.

Petrova, P. K., Cialdini, R. B., & Sills, S. J. (2007). Consistency-based compliance across cultures. *Journal of Experimental Social Psychology*, *43*(1), 104-111.

Popkin, J., & Skinner, C.H. (2003). Enhancing academic performance in a classroom serving students with serious emotional disturbance: Interdependent group contingencies with randomly selected components. *School Psychology Review, 32,* 271–284.

Pratkanis, A. R. (2007). An invitation to social influence research. *The Science of Social Influence: Advances and Future Progress*, 1-16.

Reinke, W. M., Herman, K. C., Darney, D., Pitchford, J., Becker, K., Domitrovich, C., & Ialongo, N. (2012). Using the classroom check-up model to support implementation of PATHS to PAX. *Advances in School Mental Health Promotion*, *5*(3), 220-232.

Rollnick, S., & Gobat, N. (2016). Integrating MI into services: Challenges and opportunities. *Addiction (Abingdon, England), 111*(7), 1157–1158. https://doi.org/10.1111/add.13332

Rollnick, S., & Miller, W. R. (1995). What is motivational interviewing? *Behavioural and Cognitive Psychotherapy*, *23*(04), 325-334.

Schreck, K. A., & Richdale, A. L. (2011). Knowledge of childhood sleep: A possible variable in under or misdiagnosis of childhood sleep problems. *Journal of Sleep Research*, *20*(4), 589-597.

Spangenberg, E. R., & Greenwald, A. G. (2001). Self-prophecy as a behavior modification technique in the United States. *The Practice of Social Influence in Multiple Cultures*, 51-62.

Tanner-Smith, E. E., & Lipsey, M. W. (2015). Brief alcohol interventions for adolescents and young adults: A systematic review and meta-analysis. *Journal of Substance Abuse Treatment, 51*, 1-18.

Walton, G. M., & Cohen, G. L. (2007). A question of belonging: Race, social fit, and achievement. *Journal of Personality and Social Psychology*, *92*(1), 82.

Walton, G. M., & Cohen, G. L. (2011). A brief social-belonging intervention improves academic and health outcomes of minority students. *Science, 331*, 1447–1451.

York, B, & Loeb, S. (2014). One step at a time: The effects of an early literacy text messaging program for parents of preschoolers. NBER Working Paper 20659. National Bureau of Economic Research.
