# Supplementary material for: A Cluster Randomised Trial of a School‐Based Universal Intervention Program for Middle School Students' Sleep and Related Outcomes
Source: J Sleep Res. 2025 Jun 18;35(1):e70123. doi: 10.1111/jsr.70123 (PMC12856133; doi:10.1111/jsr.70123)
Supplement: Supplementary file 3 — Data S3. [file JSR-35-e70123-s002.docx]

# Supplemental file 3

# Measures of Outcomes and Intervention Fidelity of SLEEPS

## Adolescents' Beliefs About Sleep Hygiene

Adolescents rated each item on a 4-point Likert scale (0 = “not at all true”, 1 = “hardly true”, 2 = “moderately true”, 3 = “very true”).

1. I can explain why sleep is important.
2. I can describe the effects of kids not getting enough sleep.
3. I can describe actions a person could take to get better sleep.
4. I can list barriers that get in the way of sleep.

## Mechanisms of Change Factors Based on the TPB

Adolescents rated each item on a 4-point Likert scale (1 = “Not at all true”, 2 = “To a small degree this is true”, 3 = “To a moderate degree this is true”, 4 = “To a large degree this is true", 5 = “Completely true”).

*Attitude* *Scale*

1. Getting the sleep I need leads to good things for me.
2. People who focus on getting good sleep are better off than those who do not.
3. I feel that getting good sleep is important to be the best version of myself.

*Self-Efficacy Scale*

1. I am confident I can get good sleep if I try.
2. I have control over doing things to get good sleep.
3. I have the ability to get good sleep even when things come up that make it harder.

*Subjective Norms Scale*

1. Other students my age believe it's important to get good sleep.
2. Other students my age focus on getting good sleep to be the best possible version of themselves.
3. Adults in my life expect me to get good sleep to be healthy and perform to the best of my abilities.

*Behavioral Intention* *Scale*

1. I intend to put in the effort to get good sleep.
2. Focusing on getting good sleep is a high priority for me.
3. I will try my best to get good sleep.

## SLEEPS Fidelity Checklist: Lesson xx

| **Directions**: This questionnaire is about the implementation of the *SLEEPS* Curriculum. Read the statements and select the response that best reflects your opinion. Please answer each question carefully. Please do not skip any items. |
| --- |

**Each lesson contains a Warm-Up, Interactive Group (small and whole group) Discussions, Individual Reflections (motivational interviewing strategies), Story and Discussion, Assignment of Homework, and Wrap-Up, and Dissemination of Caregiver Resources. Please check which of these you were able to complete for Lesson xx.**

___ Warm up

___ Interactive Group Discussions

___ Story and Discussion

___ Assignment of homework

___Wrap-Up

___Dissemination of Caregiver Resources

| **ADHERENCE** | **Never** | **Occasionally** | **Often** | **Always** |
| --- | --- | --- | --- | --- |
| When teaching lessons, people sometimes skip or change parts of the lessons. To what extent did you do the following for Lesson X? |  |  |  |  |
| Leave out parts of the lesson |  |  |  |  |
| Skip parts of the lesson |  |  |  |  |
| Change the lesson **significantly** from the way it was written |  |  |  |  |
| Add new material to the lesson |  |  |  |  |
| Did you send out the Caregiver Resource activities for Lesson xx | ___Yes ___No ___Not Applicable | | | |

| **EXPOSURE DOSAGE** | **100%** | **90%** | **80%** | **70%** | **60%** | **50%** | **Less than 50%** |
| --- | --- | --- | --- | --- | --- | --- | --- |
| Of all the students in the class, what proportion of them received the lesson? |  |  |  |  |  |  |  |
| Approximately what percentage of your students seemed engaged with Lesson X? |  |  |  |  |  |  |  |

| **COMPETENCY** | **Never** | **Occasionally** | **Often** | **Always** |
| --- | --- | --- | --- | --- |
| While I taught the lesson, when my students were participating in activities, I offered praise or positive recognition for their effort. |  |  |  |  |
| While I taught the lesson, my students asked and answered questions that were on topic*.* |  |  |  |  |
| While I taught the lesson, my students used the skills they learned from **Lesson x** effectively *when prompted.* |  |  |  |  |

| **STUDENT RESPONSIVENESS** | **Not at all** | **A little** | **Some** | **A lot** |
| --- | --- | --- | --- | --- |
| To what extent were students distracting other students during **Lesson X**? |  |  |  |  |
| To what extent were you able to manage student behavior during **Lesson X**? |  |  |  |  |
| To what extent were your students following along with **Lesson X**? |  |  |  |  |
| To what extent were students participating in group and individual reflective activities? |  |  |  |  |
