# Supplementary material for: A Cluster Randomised Trial of a School‐Based Universal Intervention Program for Middle School Students' Sleep and Related Outcomes
Source: J Sleep Res. 2025 Jun 18;35(1):e70123. doi: 10.1111/jsr.70123 (PMC12856133; doi:10.1111/jsr.70123)
Supplement: Supplementary file 5 — Data S5. [file JSR-35-e70123-s005.docx]

# Supplemental file 5: Multilevel Model Equations

*Note.* i= student ID, j= teacher ID. There are 12 proximal and distal Outcome_ij_, including

**Five mechanisms of change factors**: *beliefs, attitude, subjective norm, self-efficacy, behavioral intention* *about enacting healthy sleep behaviors*, **three types of sleep behaviors**: *Sleep Routine and Environment, Technology Use During Bedtime, Sleep-Related Cognition and Emotion*, **one indicator of** *sleep quality as daytime sleepiness*, **one indicator of** *internalizing symptoms*, **Two academic enablers**: *academic motivation* and *school engagement*.

**RQ1: Main Effect Models**

**Level 1 Equation: student-level (n = 104)**

Outcome_ij_ = ${\beta_{0}+\beta}_{1}*\left( Student Age \right)+\beta_{2}*\left( \mathrm{Gender} \right)+\beta_{3}*\left( \mathrm{FRPL} \right)+\beta_{4}*\left( \mathrm{Race} \right)+\beta_{5}*\left( Baseline Outcome \right)$ + $r_{\mathrm{ij}}$

**Level 2 Equation: teacher/classroom level (n = 8)**

$\beta_{0}$ = $\gamma_{00}$ +$\gamma_{01}*\left( \mathrm{Treatment} \right)+ \gamma_{02}*\left( Teaching Experience \right)$+ $\mu_{0j}$

$\beta_{1}$ = $\gamma_{10}$

$\beta_{2}$ = $\gamma_{20}$

$\beta_{3}$ = $\gamma_{30}$

$\beta_{4}$ = $\gamma_{40}$

$\beta_{5}$ = $\gamma_{50}$

**RQ2: Cross-level Interaction Between Treatment and Baseline Outcome**

**Level 1 Equation: student-level (n = 104)**

Outcome_ij_ = ${\beta_{0}+\beta}_{1}*\left( Student Age \right)+\beta_{2}*\left( \mathrm{Gender} \right)+\beta_{3}*\left( \mathrm{FRPL} \right)+\beta_{4}*\left( \mathrm{Race} \right)+\beta_{5}*\left( Baseline Outcome \right)$ + $r_{\mathrm{ij}}$

**Level 2 Equation: teacher/classroom level (n = 8)**

$\beta_{0}$ = $\gamma_{00}$ +$\gamma_{01}*\left( \mathrm{Treatment} \right)+ \gamma_{02}*\left( Teaching Experience \right)$+ $\mu_{0j}$

$\beta_{1}$ = $\gamma_{10}$

$\beta_{2}$ = $\gamma_{20}$

$\beta_{3}$ = $\gamma_{30}$

$\beta_{4}$ = $\gamma_{40}$

$\beta_{5}$ = $\gamma_{50}$ +$\gamma_{51}*\left( \mathrm{Treatment} \right)$

**RQ 3: Cross-level Interaction Between Treatment and Student Demographics**

**Level 1 Equation: student-level (n = 104)**

Outcome_ij_ = ${\beta_{0}+\beta}_{1}*\left( Student Age \right)+\beta_{2}*\left( \mathrm{Gender} \right)+\beta_{3}*\left( \mathrm{FRPL} \right)+\beta_{4}*\left( \mathrm{Race} \right)+\beta_{5}*\left( Baseline Outcome \right)$ + $r_{\mathrm{ij}}$

**Level 2 Equation: teacher/classroom level (n = 8)**

$\beta_{0}$ = $\gamma_{00}$ +$\gamma_{01}*\left( \mathrm{Treatment} \right)+ \gamma_{02}*\left( Teaching Experience \right)$+ $\mu_{0j}$

$\beta_{1}$ = $\gamma_{10}$ +$\gamma_{11}*\left( \mathrm{Treatment} \right)$

$\beta_{2}$ = $\gamma_{20}$ +$\gamma_{21}*\left( \mathrm{Treatment} \right)$

$\beta_{3}$ = $\gamma_{30}$ +$\gamma_{31}*\left( \mathrm{Treatment} \right)$

$\beta_{4}$ = $\gamma_{40}$ + $\gamma_{41}*\left( \mathrm{Treatment} \right)$

$\beta_{5}$ = $\gamma_{50}$
